# Set2 family regulates mycotoxin metabolism and virulence via H3K36 methylation in pathogenic fungus *Aspergillus flavus*

**DOI:** 10.1080/21505594.2022.2101218

**Published:** 2022-08-09

**Authors:** Zhenhong Zhuang, Xiaohua Pan, Mengjuan Zhang, Yaju Liu, Chuanzhong Huang, Yu Li, Ling Hao, Shihua Wang

**Affiliations:** a Key Laboratory of Pathogenic Fungi and Mycotoxins of Fujian Province, Key Laboratory of Biopesticide and Chemical Biology of Education Ministry, and School of Life Sciences, Fujian Agriculture and Forestry University, Fuzhou, China; b Immuno-Oncology Laboratory of Fujian Cancer Hospital, Fujian Provincial Key Laboratory of Translational Cancer Medicine, Fujian Medical University Cancer Hospital, Fuzhou, China; c Fujian Key Laboratory of Propagated Sensation along Meridian, Fujian Academy of Chinese Medical Sciences, Fuzhou, China

**Keywords:** *Aspergillus flavus*, histone methyltransferase, Set2 family, virulence, mycotoxin

## Abstract

*Aspergillus flavus* infects various crops with aflatoxins, and leads to aspergillosis opportunistically. Though H3K36 methylation plays an important role in fungal toxin metabolism and virulence, no data about the biological function of H3K36 methylation in *A. flavus* virulence has been reported. Our study showed that the Set2 histone methyltransferase family, AshA and SetB, involves in morphogenesis and mycotoxin anabolism by regulating related transcriptional factors, and they are important for fungal virulence to crops and animals. Western-blotting and double deletion analysis revealed that AshA mainly regulates H3K36me2, whereas SetB is mainly responsible for H3K36me3 in the nucleus. By construction of domain deletion *A. flavus* strain and point mutation strains by homologous recombination, the study revealed that SET domain is indispensable in mycotoxin anabolism and virulence of *A. flavus*, and N455 and V457 in it are the key amino acid residues. ChIP analysis inferred that the methyltransferase family controls fungal reproduction and regulates the production of AFB1 by directly regulating the production of the transcriptional factor genes, including *wetA*, *steA*, *aflR* and amylase, through H3K36 trimethylation in their chromatin fragments, based on which this study proposed that, by H3K36 trimethylation, this methyltransferase family controls AFB1 anabolism through transcriptional level and substrate utilization level. This study illuminates the epigenetic mechanism of the Set2 family in regulating fungal virulence and mycotoxin production, and provides new targets for controlling the virulence of the fungus *A. flavus*.

AUTHOR SUMMARY

The methylation of H3K36 plays an important role in the fungal secondary metabolism and virulence, but no data about the regulatory mechanism of H3K36 methylation in the virulence of *A. flavus* have been reported. Our study revealed that, in the histone methyltransferase Set2 family, AshA mainly catalyzes H3K36me2, and involves in the methylation of H3K36me1, and SetB mainly catalyzes H3K36me3 and H3K36me1. Through domain deletion and point mutation analysis, this study also revealed that the SET domain was critical for the normal biological function of the Set2 family and that N455 and V457 in the domain were critical for AshA. By ChIP-seq and ChIP-qPCR analysis, H3K36 was found to be trimethylation modified in the promotors and ORF positions of *wetA*, *steA, aflR* and the amylase gene (AFLA_084340), and further qRT-PCR results showed that these methylation modifications regulate the expression levels of these genes. According to the results of ChIP-seq analysis, we proposed that, by H3K36 trimethylation, this methyltransferase family controls the metabolism of mycotoxin through transcriptional level and substrate utilization level. All the results from this study showed that Set2 family is essential for fungal secondary metabolism and virulence, which lays a theoretical groundwork in the early prevention and treatment of *A. flavus* pollution, and also provides an effective strategy to fight against other pathogenic fungi.

## Introduction

The worldwide distributed filamentous fungus, *Aspergillus flavus*, widely contaminated a variety of important grain and oil crops (including peanut and corn) with its secondary metabolites, especially the mycotoxin aflatoxin B1 [1–3]. *A. flavus* also endangers human health through infection and pathogenic growth, and even threatens the life of immunocompromised patients by aspergillosis [4,5]. As the most toxic mycotoxin, AFB1 leads to immunosuppression, fatty liver, even liver cancer under chronic exposure, and causes aflatoxicosis, even death, under acute exposure [6–8]. The virulence and mycotoxin metabolism of the pathogenic fungus are regulated by a complex network involving multiple levels, include transcriptional level, post-translational modification level, epigenetic level [9–11]. Recently, the mechanism of the up-stream epigenetic regulation in fungal development and virulence has become one of research hotspots [12].

Epigenetic regulation induces changes in gene expression level and affects a variety of metabolic processes in response to internal and external signals during growth and development, and it is mainly regulated by three mechanisms: covalent DNA and histone modifications, and noncoding RNA [13,14]. Histone post-translational modification, regulating by relating enzymes, plays a critical role in the transcription of corresponding genes by chromatin remodelling [15]. Modifications of key amino acids on histone *N*-terminal tail are revealed as “Histone codes,” they recruit regulatory chromatin enzymes to initiate the processes of heterochromatization or euchromatization, which further directs the silence or expression of related genes, and these “Histone codes” including methylation phosphorylation, ubiquitination, acetylation, and SUMOylation [16–19]. Histone methylation has recently become a hot spot in the field of epigenetics, and it was found that several lysine residues within histones, including K4, K9, K27, K36, and K79 of histone H3, were always specifically methylated by a number of epigenetic enzymes [17,20]. It is revealed that the presence of methyl groups on the corresponding lysine leads to gene activation (such as H3K4 and H3K36), or gene repression (such as H3K27 and H3K9) [21,22]. In view of the critical roles of histone lysine methylation, it is vital to explore the bio-functions of the histone lysine methylation in the mycotoxin metabolism and virulence of pathogenic fungi.

Trithorax Group (TrxG) protein-Ash1 (absent, small, or homoeotic discs 1) is a conserved chromatin factor that contains a SET domain for histone methylation, and is involved in the transcriptional activation of many important developmental genes [23–26]. Ash1-like MoKMT2 H is required for the virulence of *Magnaporthe oryzae* to rice [27]. As a novel histone methyltransferase (HMT), Set2 catalyzes the mono-, di-, and tri-methylation of H3K36 through its SET domain in *Saccharomyces cerevisiae*, while a recent study revealed that trimethylation of H3K36 resulting from the catalysing activity of both Set2 and Ash1 (the Set2 family) [28,29]. Considering their great impact in the maintenance of euchromatic station, it is critical to clarify the biological function of Set2 family members in the secondary metabolism and virulence of pathogenic *Aspergillus spp*.

Although there have been some related studies reported on the role of lysine histone methyltransferases in the epigenetic regulation of *A. flavus*, the bio-function of the Set2 family, including Ash1 (defined as AshA in *A. flavus*) and Set2 (defined as SetB in *A. flavus*), in this pathogenic fungus is still unknown. In our previous qRT-PCR analysis in this fungus, an obvious linear correlation was found between the expression level of *ashA* and AFB1 regulator *aflR* at 28°C, which suggested that the Set2 family might play an important role in the mycotoxin metabolism in the fungus. Thus, a question raised that how the methylation of H3K36 catalysed by Set2 family could affect the secondary metabolism and virulence of filamentous fungi. This work was designed to illuminate the biological function of Set2 family in mycotoxin metabolism and virulence of *A. flavus*.

## Results

### *AshA is conserved in* Aspergillus spp. *and required in fungal morphogenesis*

AshA protein in this pathogenic fungus and the ortholog of the protein from other 15 fungal species (*A. parasiticus*, *A. oryzae*, *A. nomius*, *A. niger*, *A. kawachii*, *A. clavatus, A. luchuensis, A. fischeri*, *A. terreus*, *A. lentulus*, *A. udagawae*, *A. fumigatus*, *R. emersonii*, *T. islandicus* and *T. marneffei*) were aligned by MEGA5.1, and the result reflected that AshA in three fungal species, including *A. flavus*, *A. oryzae* and *A. parasiticus*, had the highest similarity, among which the similarity between *A. flavus* and *A. oryzae* was 100%. The lowest homology was revealed in the pathogenic fungus and three non-Aspergillus species (*R. emersonii*, *T. islandicus* and *T. marneffei*), and they were 57.4%, 52.93% and 51.64%, respectively. The phylogenetic tree was constructed and it showed that *A. flavus*, *A. oryzae* and *A. parasiticus* were classified into one cluster, and all *Aspergillus spp*. were grouped into a big cluster (Figure S1A). And a SET domain was revealed to present in all these 16 fungal species by the protein domain analysis (Figure S1B).

Through homologous recombination, the *ashA* deletion mutant (Δ*ashA*) was prepared in the study (Figure S1C), and the result transformants were confirmed by southern-blotting (Figure S1D) and PCR (Figure S1F) analysis. The southern-blot results coincided with the digestion scheme shown in Figure S1C. The *ashA* complementation strain (Com-*ashA*) was prepared, and confirmed by PCR (Figure S1F) according to the construction diagram shown in Figure S1E. Further qRT-PCR analysis revealed that the transcriptional level of *ashA* was undetectable in Δ*ashA* strain, but recovered in Com-*ashA* strain (Figure S1G). All these results showed that the Δ*ashA* and Com-*ashA* strains of the pathogenic fungus were successfully prepared.

**Figure 1.**
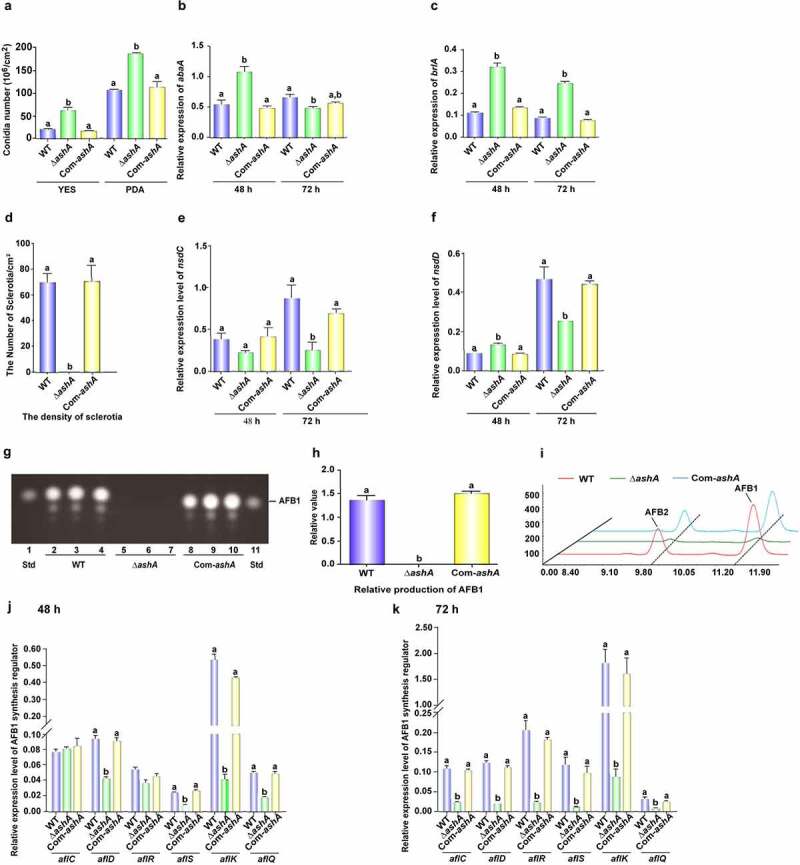
The role of AshA in the development and aflatoxin biosynthesis. (a) Conidial quantification of cultures of WT, Δ*ashA* and Com-*ashA* strains on YES agar and PDA at 37°C in the dark for 4 d. (b) Relative expression levels of *abaA* gene in the above strains in YES at 37°C at 48 h and 72 h. (c) Relative expression levels of *brlA* gene in the above strains in YES at 37°C at 48 h and 72 h. (d) Sclerotia quantification of WT, Δ*ashA* and Com-*ashA* strains on CM media at 37°C in the dark for 6 d. (e) Relative expression level of *nsdC* gene in the above strains in CM media at 37°C at 48 and 72 h. (f) Relative expression level of *nsdD* gene in the above strains in CM media at 37°C at 48 and 72 h. (g) TLC analysis of aflatoxins production from WT, Δ*ashA* and Com-*ashA* strains after 6 d of incubation in dark in liquid YES media. (h) Relative quantification of AFB1 production of WT, Δ*ashA* and Com-*ashA* strains according to the result of panel G. (i) HPLC analysis of aflatoxins from WT, Δ*ashA* and Com-*ashA* strains cultivated in liquid YES media for 6 d. (j) Relative expression levels of aflatoxin bio-synthesis and regulation genes monitored by qRT-PCR at 48 h. All *A. flavus* strains were cultivated in liquid YES media. (k) Relative expression levels of aflatoxin bio-synthesis and regulation genes at 72 h. All fungal strains were cultivated in liquid YES media.

To reveal the biological function of AshA in the fungal reproduction, above *A. flavus* strains were incubated onto YES agar and PDA media for 4 d cultivation. Δ*ashA* strain showed increased conidial production, and more conidiophores developed in Δ*ashA* strain, too (Figure 1(a)). Following, the transcriptional levels of conidiation related regulator genes *abaA* and *brlA* were detected by qRT-PCR, which revealed that the absence of AshA increased the expression level of *abaA* at 48 h (Figure 1(b)), and also significantly enhanced the expression level of *brlA* at the time points of 48 and 72 h (Figure 1(c)). These results reflected that AshA negatively regulates fungal asexual propagation on YES medium. To assess the role of AshA in the assembly of resistant structure – sclerotia, above fungal strains were point cultured onto CM at 37°C. Then, the sclerotium number was counted after the topical hyphae and conidia were removed by 70% ethanol. The result showed that no sclerotia were found in Δ*ashA* strain (Figure 1(d)). The transcriptional levels of sclerotia regulator genes, both *nsdC* and *nsdD*, were also monitored by qRT-PCR, which discovered that the expression level of *nsdC* was markedly decreased at 48 and 72 h (Figure 1(e)), and *nsdD* at 72 h was also markedly down-regulated in Δ*ashA* strain (Figure 1(f)). All these results reflect that AshA plays an important role in the sclerotia development.

The error bars represent the standard error (*P* < 0.05).

### AshA is essential for the biosynthesis of AFB1

The bio-function of AshA in the synthesis of aflatoxin was assayed by incubating the WT, Δ*ashA* and Com-*ashA* strains in liquid YES media, and the hyphae was harvested on the 6th d and analysed by TLC. The result displayed that the production of AFB1 was dramatically decreased when AshA was absent (Figure 1(g,h)). The further HPLC analysis confirmed that the Δ*ashA* strain almost lost its capability in AFB1 and AFB2 synthesis (Figure 1(i)). Following, the transcriptional levels of aflatoxin synthesis genes (including *aflC* gene, *aflD* gene, *aflK* gene, and *aflQ* gene) and transcriptional regulation genes (including both *aflR* gene and *aflS* gene) at 48 and 72 h were monitored with qRT-PCR, which discovered that the transcriptional levels of all these related genes were markedly down-regulated in Δ*ashA* at 72 h (Figure 1(j,k)). Above results proposed that AshA up-regulates aflatoxin production via positively regulating related biosynthesis gene cluster.

### *AshA plays important role in virulence of* A. flavus *host*

**Figure 2.**
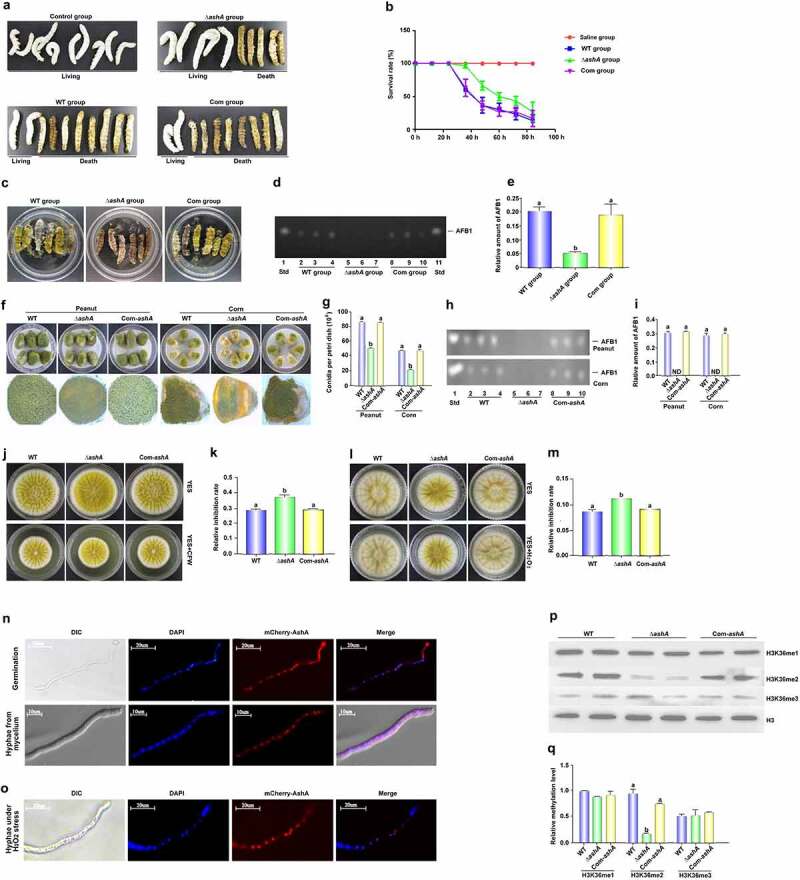
The role of AshA in the *A. flavus* virulence against silkworm and crop kernels, and its subcellular location and the methylation site and levels catalyzed by it. (a) Photographs of the silkworms infected with the WT (WT group), Δ*ashA* (Δ*ashA* group) and Com-*ashA* (Com group) of *A. flavus* strains after 1 week incubation. (b) the survival rate of silkworms 1 week after injection of above *A. flavus* strains, respectively. (c) Photographs of the dead silkworms infected by *A. flavus* after 6 d incubation in dark under 28°C. (d) TLC analysis of AFB1 levels produced in infected dead silkworms that are shown in C. (e) the histogram showing the relative amount of AFB1 in silkworms according to panel D. (f) Photographs of the peanut seeds and corn kernels infected with the *A. flavus* WT, Δ*ashA* and Com-*ashA* strains after 7 d incubation in the dark. (g) Quantification of conidia from the surface of *A. flavus* strains colonized peanut and corn grains in panel a and B. (h) TLC analysis of AFB1 levels of *A. flavus* strains colonized peanut and corn grains shown in panel F. (i) Relative amount of AFB1 in peanut and corn grains, according to the result of H. (j) Point inoculated cultures of *A. flavus* WT, Δ*ashA*, and Com-*ashA* strains in YES and YES+80 µg/ml CFW. (k) Relative inhibition rate of CFW to *A. flavus* strains on the 4th d. (l) Point inoculated cultures of *A. flavus* WT, Δ*ashA* and Com-*ashA* strains in YES and YES+5 mM H_2_O_2_ in the dark for 4 d. (m) the histogram showing relative inhibition rate of H_2_O_2_ to above *A. flavus* strains on the 4th d. (n) Maps of the germinating spore and hyphae shown by DIC imaging, the location of the nucleus with DAPI staining (Exciting wavelength: 405 nm UV light. Emission wavelength: 420–460 nm), the location of mCherry-AshA (Exciting wavelength: 552 nm. Emission wavelength: 600–630 nm), and merged photo of nucleus and mCherry-AshA. The mCherry-AshA was expressed under the promotor of *gpdA*(*p*). (o) Maps of hyphae under the stress of H_2_O_2_ with DIC imaging, DAPI staining, mCherry-AshA fluorescence imaging, and merged photo of DAPI staining and fluorescence imaging. (p) Western-blotting analysis on the bio-function of AshA in methylation level of H3K36me (1–3). (q) the histogram showing the relative 1–3 methylation level of H3K36 according to the results of western-blotting analysis in panel P according to the size and density of bands of H3K36me (1–3), respectively.

To investigate the role of AshA in fungal virulence to insects, silkworms were injected with 5 µL (10^6^/mL) conidia suspension from *A. flavus* WT, Δ*ashA* and Com-*ashA* strains, and kept at 25°C for 84 h. It was found that the survival rate of silkworms injected by spores of Δ*ashA* strain was significantly higher than the silkworms injected with spores from WT and Com-*ashA* fungal strains (Figure 2(a,b)). Dead silkworms from WT, Δ*ashA* and Com-*ashA* injection groups were collected and further inoculated for 6 d in dark under 28°C. Besides obviously more conidia were found on the surface of the dead silkworms from the WT and Com-*ashA* injection groups (Figure 2(c)), TLC analysis showed that *ashA* mutant significantly repressed the synthesis capacity of AFB1 in *A. flavus* contaminated silkworms (Figure 2(d,e)). Above results revealed that AshA plays an essential role in *A. flavus* virulence to insects.

Relative inhibition rate = (diameter of the colony without inhibitor - diameter of the colony with inhibitor)/diameter of the colony without inhibitor. The error bars represent the standard error (*P* < 0.05).

The kernels of maize and peanut were incubated with conidia of *A. flavus* strains for 7 d, and the result (Figure 2(f,g)) displayed that conidia production from the Δ*ashA* incubated kernels was significantly reduced compared to those from the WT fungal strain on the kernels of both peanut and corn. By TLC assay, it was found that the synthesis capacity of AFB1 of this pathogenic fungus on both kernels was dramatically repressed after *ashA* was deleted (Figure 2(h,i)). All above results indicated that AshA plays important role in *A. flavus* virulence to crops.

In view of all above results coming from the silkworms and crop kernels infection models, we speculated that the absence of AshA may improve the sensitivity of the Δ*ashA* fungal strain to some stress factors in organisms, so the fungal strains, including *A. flavus* WT, Δ*ashA*, and Com-*ashA* were inoculated onto YES agar with 0.01% MMS, 80 µg/mL CFW, 0.03% SDS, 0.5% CR, 10 mM HU, or 5 mM H_2_O_2_. When strains were incubated with CFW, the inhibition rate to Δ*ashA* strain was remarkedly greater than those to WT and Com-*ashA* fungal strains at 4th d (Figure 2(j,k)), showing that *ashA* deletion mutant was more susceptible to cell wall formation inhibitor. When the above *A. flavus* strains were inoculated with H_2_O_2_, the inhibition effect of H_2_O_2_ to Δ*ashA* was significantly stronger, too (Figure 2(l,m)). To confirm whether AshA plays a role in the sensitivity of *A. flavus* to CFW and H_2_O_2_ mediated oxidative stress, the fungal strains were grown on a series of relative stress gradient PDA plates (CFW: from 40 to 400 µg/mL; H_2_O_2_: from 3 to 6 mM), and the results showed that in lower concentration (from 40 to 80 µg/mL), the lack of AshA increases the sensitivity of *A. flavus* to the cell wall inhibitor (Figure S2A and S2B); To H_2_O_2_, there was no significant difference in the growth of WT and Δ*ashA* strains in lower concentration (3 to 4 mM) of it, while in the higher concentration (5 to 6 mM) of H_2_O_2_, AshA is obviously involved in the stress response to H_2_O_2_ (Figure S2C and S2D), which reflected that the deletion of *ashA* significantly enhances fungal sensitivity to H_2_O_2_ mediated oxidative stress. But no obvious evidence showed that AshA is involving the sensitivity of the fungal to MMS, SDS, CR or HU (data no shown). All above results indicated that AshA plays a critical role under the stress of CFW and H_2_O_2_, and it may improve fungal virulence in the host by improving fungal cell wall resistance and fungal antioxidant effect.

### AshA regulates H3K36me2 in the nucleus

To explore the subcellular location of AshA, we prepared a *A. flavus* strain (*mCherry-ashA*), in which a mCherry tag was expressed at AshA *N*-terminus. The *mCherry-ashA* strain was inoculated on YES culture, and the mycelium was collected at 4 h (observing spore germination) and 12 h (observing hyphae), then incubated in DAPI (1 μg/mL) for another 15 min. The sample was observed with the confocal laser scanning microscope (Leica SP8), which showed that most mCherry:AshA accumulated in the nucleus of each cell in both germination and hyphal growth stage (Figure 2(n)). To examine if AshA would translocate under stresses, the *mCherry-ashA* strain was also incubated with 5 mM H_2_O_2_ for 12 h, and the results reflected that AshA mostly accumulated in the nucleus even under oxidative stress (Figure 2(o)).

The enzymatic function of AshA as a methyltransferase was analysed with western-blotting analysis. The protein samples prepared from *A. flavus* strains were transferred onto NC membrane and detected with the antibodies against 1 to 3 methylation of H3K4, H3K36 and H3K79. The results showed that when *ashA* was deleted, only few H3K36me2 was detected (Figure 2(p,q)), indicating that AshA mainly regulates the product of H3K36me2. Further western-blotting assay demonstrated that AshA did not involve in the methylation of H3K4 and H3K79 (data no shown). Above results indicated that AshA mainly catalyzes H3K36me2.

### SET domain and N455 and V457 in it play an important role in fungal virulence

To explore the role of SET domain in the bio-function of AshA, the SET domain mutant (*ashA*^*ΔSET*^) was constructed by homologous recombination (Figure S3B) and confirmed by sequencing (data no shown). The mutants were inoculated on YES medium at 37°C, and the result showed that, similar to the result from Δ*ashA* strain, the *ashA*^*ΔSET*^ mutant produced dramatically more conidia (Figure S3C and S3D), but no sclerotia formed at 37°C for 7 d incubation (Figure S3E and S3F). These results revealed that the SET domain plays critical role in the development of *A. flavus*. These *A. flavus* strains were grown in liquid YES for aflatoxin production, and the TLC results showed similar results to Δ*ashA*, and no AFB1 could be observed in *ashA*^*ΔSET*^ strain (Figure S3G and S3H). To explore if the deletion of SET domain affects the expression level of *ashA*, the expression level of *ashA* in *ashA*^*ΔSET*^ mutant was compared with that of WT strain, and the results confirmed that the absence of SET domain doesn’t affect *ashA* expression obviously (Figure S3I). To test the role of SET domain in the virulence of *A. flavus*, the whole living crop kernel was mixed with 10^5^/mL spores from each *A. flavus* strains, then the cultures were kept at 28°C for a week (Figure S4A). These results displayed that the conidiation capacity of *ashA*^*ΔSET*^ mutant was dramatically down-regulated on the kernels of both living peanut and corn (Figure S4B and S4C), and no AFB1 was observed in the results of TLC analysis, compared to WT strain (Figure S4D to S4F). All these results showed that SET domain is the core structural element of AshA.

In exploring the key amino acids which play a critical role in the SET domain, the conserved N455 and V457 in the domain were located by Clustal X and further characterized with MEME (Figure S3A). To study the bio-function of the amino acids N455 and V457, the uncharged polar Asparagine 455 was replaced by non-polar alanine, and the hydrophobic non-polar valine 457 was replaced by hydrophilic polar aspartic acid in the point mutant strains (*ashA*^N455A^ and *ashA*^V457D^) by homologous recombination, and confirmed by sequencing (data no shown). The mutants were inoculated on YES media, it was found that the conidiation capacity of both point mutants dramatically increased, but the sclerotia number was significantly decreased in *ashA*^N455A^ mutant, and none was formed in *ashA*^V457D^ mutant after 7 d incubation on the YPD medium (Figure S3C to S3F). The results reflected that N455 and V457 in the domain play essential roles in fungal propagation. The TLC analysis result displayed that the amount of AFB1 produced by the above two point-mutants was significantly lower compared to that of the WT strain shown in Figure S3G and S3H. The expression levels of *ashA* in *ashA*^N455A^ and *ashA*^V457D^ mutants were also compared with that of WT strain, and the results showed that the mutation of N455 or V457 doesn’t affect *ashA* expression, too (Figure S3I). To test their role in fungal virulence, the whole living crop kernel was mixed with 10^5^/mL spores, and the cultures were kept at 28°C for a week (Figure S4A). The results showed that the conidiation and AFB1 bio-synthesis capacity of *ashA*^N455A^ and *ashA*^V457D^ was significantly down-regulated on both living peanut and corn kernels (Figure S4B to S4F). All these results showed that both N455 and V457 are important for the bio-function of SET domain.

### SetB mainly catalyzes the trimethylation of H3K36 in the nuclei

The set2 methylation transferase family includes Ash1 and set2 (Corresponding to AshA and setB in *A. flavus*) [29]. In view of the critical regulatory function of AshA in fungal reproduction and virulence as presented above, in order to reveal the role of the whole set2 family in *A. flavus*, this study further explored the bio-function of SetB. By bioinformatics analysis, we found that, similar to AshA, SetB is conservative among fungi, especially among *Aspergillus spp*. (Figure S5A and S5B). To illuminate the biological function of SetB in the development and virulence of this pathogenic fungus, we constructed both *setB* deletion strain (*ΔsetB*) and complementary strain (Com*-setB*). The construction scheme and the confirmation of the above strains by southern-blotting, diagnostic PCR and qRT-PCR were given in Figure S5C to S5G. To reveal the bio-function of SET domain in SetB, we constructed a SET domain deletion strain (setB^*ΔSET*^). To explore the synergy between AshA and SetB, we further constructed *ashA* and *setB* genes double deletion strain (*ΔsetB/ashA*). H3K36A point mutant (H3K36A) was also constructed to illuminate the methylation targets of Set2 family. The constructed above fungal strains were verified through sequencing by BioSune (Shanghai, China) and diagnostic PCR (data no shown).

**Figure 3.**
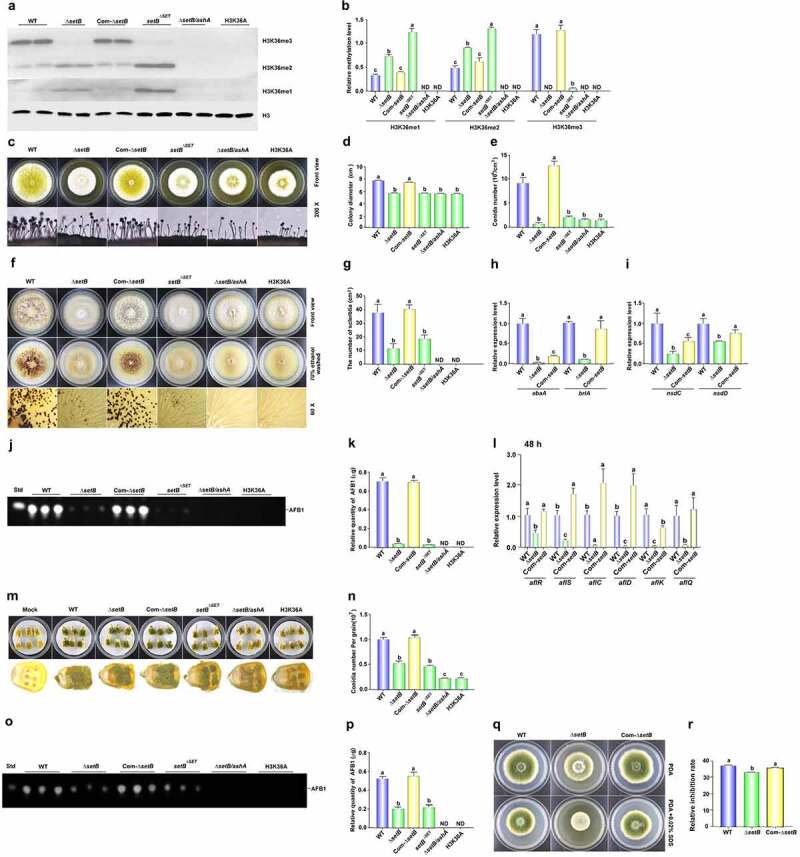
The bio-function of SetB in the methylation of H3K36, and the role of SetB in the development, AFB1 biosynthesis and virulence of *A. flavus*. (a) Western-blotting analysis on the bio-function of *setB* gene, *SET* domain, and the interaction of *setB* and *ashA* genes in the methylation of H3K36. (b) the histogram showing the relative 1–3 methylation levels of H3K36 according to the results of western-blotting analysis. (c) the fungal strains, including Δ*setB*, WT, Com*-setB*, setB^*ΔSET*^,*ΔsetB/ashA*, and H3K36A, were inoculated on PDA media under 37°C in the dark for 4 d. (d) Colony diameter comparison among Δ*setB*, WT, *δsetb-Com*, setB^*ΔSET*^,*ΔsetB/ashA*, and H3K36A fungal strains. (e) the comparison of conidia numbers produced by above fungal strains on PDA media under 37°C for 4 d. (f) the sclerotia were formed by WT, Δ*setB* and Com*-setB* strains on CM media under 37°C in the dark for 7 d. (g) Histogram showing the statistical analysis results on the role of SetB, SET domain, and the interaction of *S*etB and AshA in sclerotia formation. (h) Expression levels of *abaA* and *brlA* genes among Δ*setB*, WT, Com*-setB* strains at 48 h. The fungal strains were inoculated in PDB media under 37°C in the dark. (i) Relative expression level of *nsdC* and *nsdD* genes among Δ*setB*, WT, Com*-setB* strains at 48 h. The fungal strains were inoculated in liquid CM media under 37°C in the dark. (j) the productions of AFB1 were monitored by TLC after above strains cultured in liquid YES media under 28°C in the dark for 6 d. (k) the relative mycotoxin productions were shown with histogram according to the results from panel J. (l) qRT-PCR analysis was carried out on orthodox AFB1 biological synthesis pathway genes after above fungal strains were cultured in liquid YES media under 28°C for 48 h. (m) the colonization of *setB*, WT, *δsetb-Com*, setB^*ΔSET*^,*ΔsetB/ashA*, and H3K36A fungal strains on the surface of maize kernels after 7 d incubation under 28°C. (n) the histogram showing the amount of conidia produced on the surface of maize kernels. (o) TLC analysis of AFB1 produced on these maize kernels colonized by above fungal strains. (p) the histogram showing the production of AFB1 in these *A. flavus* infected host kernels, according to the result from panel O. (q) Point inoculated cultures of *A. flavus* WT, Δ*setB*, and Com-*setB* strains in PDA+0.02% SDS under 37°C in the dark for 4 d. (r) Relative inhibition rate of SDS to *A. flavus* WT, Δ*setB*, and Com-*setB* strains on the 4th d. Relative inhibition rate = (diameter of the colony without inhibitor - diameter of the colony with inhibitor)/diameter of the colony without inhibitor.

To reveal the catalytic function of SetB in the methylation of H3K36, all methylation levels of H3K36 in WT, *ΔsetB, Com-ΔsetB*, setB^*ΔSET*^,*ΔsetB/ashA*, and H3K36A fungal strains were monitored with western-blotting analysis. The result showed that almost no H3K36me3 could be detected when *setB* gene was deleted (Figure 3(a)), which reflected that SetB catalyse most of H3K36me3. The same result was observed when SET domain was absent, and this result inferred that it is the key element in catalysing H3K36me3. Compared to WT strain, obviously more H3K36me1 and me2 were detected in both *ΔsetB* and setB^*ΔSET*^ strains, reflecting that H3K36me1 and me2 were accumulated due to they can’t be converted into H3K36me3 in time. When both *setB* and *ashA* genes were deleted, all methylation levels of H3K36 were abolished, just like what happened in point mutant strain H3K36A (Figure 3(a,b)), in which no methylation could happen because the methylation position lysine 36 has been mutated to alanine. The results showed that these two Set2 family belonging methyltransferases are responsible for the methylation of H3K36 in *A. flavus*. The *SetB-mCherry* strain was constructed (Figure S6A), and the results showed that most SetB:mCherry were accumulating in the nuclei in the germination stage and hyphal growth stage (Figure S6B), and this situation is quite stable even under SDS stress (Figure S6C).

The error bars represent the standard error (*P* < 0.05).

### The role of SetB in fungal virulence

To assess the roles of SetB in the growth of this pathogenic fungus, WT, *ΔsetB* and Com*-setB* strains were point-incubated on the media of PDA. After 4 d culture under 37°C, the conidiation capacity of *ΔsetB* was found to be severely repressed, and its conidiophores became thinner (Figure 3(c,e)). A similar changing trend was found in the colony diameters among these fungal strains (Figure 3(d)), which revealed that SetB is of importance in fungal sporulation and mycelial growth. To investigate the pathway, through which SetB controls the conidia production, qRT-PCR was carried out, and the result displayed that the transcriptional levels of *abaA* and *brlA* significantly decreased when *setB* was deleted (Figure 3(h)), which suggested that SetB up-regulates fungal asexual reproduction by these transcriptional factors. After 7 d cultivation on CM media, it was found that the sclerotia number of *ΔsetB* strain severely decreased (Figure 3(f,g)), which inferred that SetB is critical for sclerotia formation. Further qRT-PCR analysis inferred that SetB regulates the formation of sclerotia by NsdC and NsdD regulated orthodox sclerotia formation pathway (Figure 3(i)).

To further investigate the function of SetB in mycotoxins metabolism, the AFB1 production in these *A. flavus* strains was assayed by TLC analysis. And the results showed that almost no AFB1 from *ΔsetB* strain was detected (Figure 3(j,k)). Further qRT-PCR was implemented on the AFB1 synthesis related genes, which revealed that SetB regulates AFB1 biosynthesis by aflatoxin biosynthesis regulators, including both AflR and AflS, and the catalysing enzymes, including AflC, AflD, AflK and AflQ (Figure 3(l)).

To assess the function of SetB in fungal virulence, maize kernels were infected with the above fungal strains. The results showed that mycelium and spores produced on the corn kernels contaminated by *ΔsetB* were significantly less (Figure 3(m)). After calculating by haemocytometer, it confirmed that the absence of SetB remarkably decreased the conidiation capacity of *A. flavus* on the surface of maize kernels (Figure 3(n)). Further extraction of AFB1 with chloroform from these fungal contaminated maize kernels and analysis by TLC illustrated that yield of AFB1 in *ΔsetB* inoculated kernels was significantly decreased (Figure 3(o,p)). By stress analysis, we also found that SetB is involved in membrane stress response mediated by SDS (Figure 3(q,r)). All the above results reflected that SetB is deeply participated in the regulation of the development, mycotoxin metabolism and the colonization of plant kernels of this pathogenic fungus.

### SET domain of SetB involves in fungal virulence

SET domain is the site by which methyltransferase catalyzes lysine methylation in histone. To reveal the role of SET domain in SetB, the related fungal strains were point-inoculated on PDA agar. And the observed results displayed that the sporulation capacity and the colony diameter of setB^*ΔSET*^ were similar to that of Δ*setB* (Figure 3(c–e)), which reflected that it is important for conidiation and hyphal growth of the fungus. Like what happened in *ΔsetB*, the number of sclerotia of setB^*ΔSET*^ severely decreased (Figure 3(F)). By TLC analysis, it showed that just trace of AFB1 was detected when the SET domain was absent (Figure 3(j)), suggesting that SetB regulates AFB1 synthesis mainly through its SET domain. Mycelia and spores developed on the maize kernels inoculated with setB^*ΔSET*^ strain were significantly less compared with WT strain (Figure 3(m,n)), and obviously fewer AFB1 was found in setB^*ΔSET*^ strain inoculated kernels (Figure 3(o,p)). These results reflected that the SET domain is the key element of SetB.

### The synergetic effect of SetB and AshA in fungal development, mycotoxin metabolism and fungal virulence

To explore the role of the interaction of SetB and AshA in fungal virulence, double deletion strain *ΔsetB/ashA* was constructed. The phenotype analysis showed that *ΔsetB/ashA* produced very few spores, and the colony diameter of *ΔsetB/ashA* is significantly smaller compared with WT strain (Figure 3(c–e)). This study also found that no sclerotia was formed in *ΔsetB/ashA*, which inferred that SetB cooperates with AshA in responsible for sclerotia formation (Figure 3(f,g)). Further TLC analysis showed that no AFB1 was detected from *ΔsetB/ashA* (Figure 3(j,k)), which revealed that the interaction of SetB and AshA is indispensable in AFB1 biosynthesis.

The role of the Set2 methyltransferase family in the virulence of *A. flavus* was also assessed in this study. The above fungal strains were colonized on maize kernels, and the result demonstrated that mycelium and spores developed on the surface of the kernels contaminated by *ΔsetB/ashA* were severely decreased than those from *ΔsetB* or setB^*ΔSET*^ strain (Figure 3(m,n)). Following TLC analysis showed that no AFB1 could be detected from maize kernels contaminated by *ΔsetB/ashA* (Figure 3(o,p)). These results reflected that SetB cooperates with AshA, and the Set2 family plays a critical role in fungal virulence and AFB1 synthesis of *A. flavus* on crop kernels.

### The methylation of H3K36 regulates mycotoxin metabolism and fungal virulence

The result in Figure 3(a) showed that double deletion of AshA and SetB inhibited all methylation levels of H3K36. To assess the effects of methylation of H3K36 to the virulence and AFB1 production of *A. flavus*, H3K36 was point mutated into H3K36A (H3K36A strain), the result showed that all methylation levels of H3K36 are totally inhibited in the H3K36A strain as what happened in the *ΔsetB/ashA* strain (Figure 3(a)). And similar to what found in *ΔsetB/ashA*, spore stalks in H3K36A strain were very sparse, and produced very few spores, and its colony diameter was the same to that of *ΔsetB/ashA*, too (Figure 3(c–e)). The results reflected that the methylation of H3K36 is deeply involved the regulating of fungal conidiation and hyphal growth. No sclerotium was found in H3K36A strains, which inferred that the methylation of H3K36 is indispensable in sclerotia formation (Figure 3(f,g)). The TLC analysis showed that no AFB1 was detected from the H3K36A strain as shown in Figure 3(j,k), which revealed that the methylation of H3K36 is indispensable for AFB1 bio-synthesis.

The role of H3K36 methylation in the fungal virulence was also assessed. The fungal strains were colonized on maize kernels, and the results showed severe decrease of both mycelium and spores produced on the surface of the kernels contaminated by H3K36A strains, just similar to that of *ΔsetB/ashA* contaminated kernels (Figure 3(m,n)), and no AFB1 could be detected from maize kernels contaminated by H3K36A strain (Figure 3(o,p)). These results reflected that methylation of H3K36 is closely related to fungal virulence and mycotoxin anabolism of *A. flavus* on crop kernels.

### *Set2 family regulates trimethylation of H3K36 across the whole genome of* A. flavus

**Figure 4.**
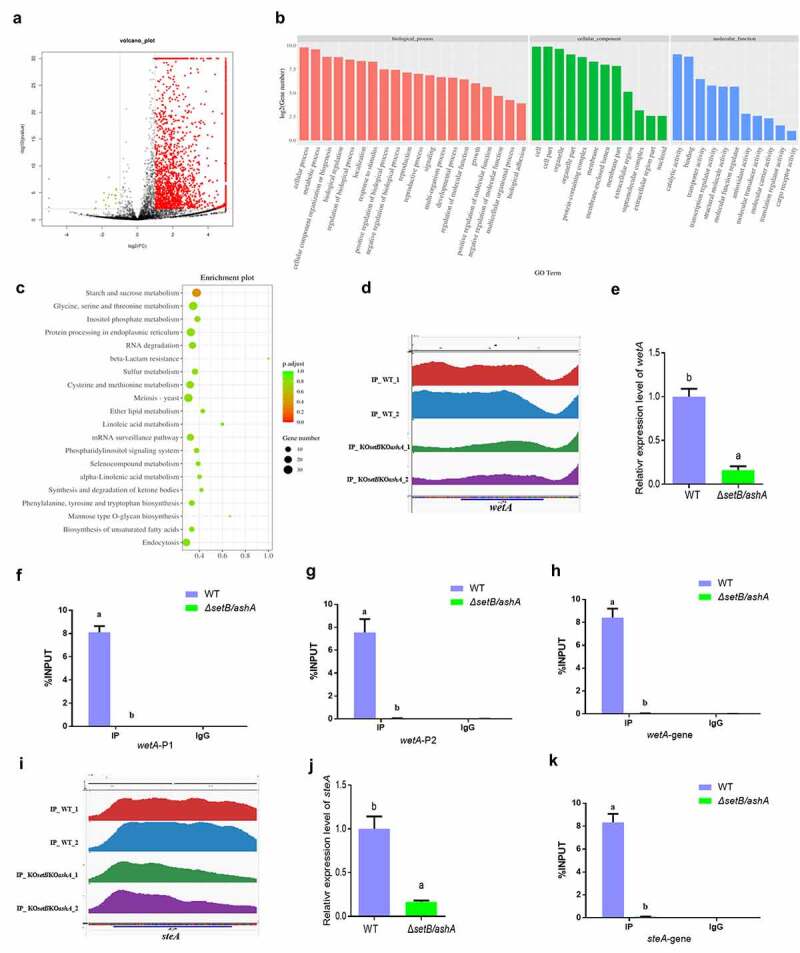
H3k36me3 catalyzed by SetB and AshA regulates many important biological processes. (a) Distributions of log2 FC (WT/*ΔsetB/ashA*), and 1995 DAPs for all 5592 peaks are significantly accumulated in WT sample (Red color; -log2 FC ≥1, *p* < 0.01), 23 DAPs are accumulated in the sample from δsetb/asha strain (green color; log2 FC ≤-1, *p* < 0.01), and 3574 peaks are not differentially regulated (black color; log2 FC < 1 or > −1, p>0.01 if log2 FC > 1 or < −1). (b) the annotation of biological processes, cellular components and molecular function for the genes which are up-regulated at WT strain compared to δsetb/asha strain. (c) the bubble graph of KEGG pathways of up-regulated gene fragments in the WT strains compared to δsetb/asha strain. (d) Comparation of the enriching levels of the H3K36me3 modified promotor and coding sequence of *wetA* gene between WT and *δsetb/asha* strain through ChIP-seq analysis. (e) qRT-PCR analysis on the expression level of the sporulation regulating gene *wetA*. (f) ChIP-qPCR analysis on the H3K36me3 modified promotor of *wetA* (−529 to −427 bp upstream of the TSS, IgG was used as negative control). (g) ChIP-qPCR analysis on second H3K36me3 modified promotor position for *wetA* (−373 to −207 bp upstream of the TSS). (h) ChIP-qPCR analysis on the H3K36me3 modified gene coding sequence (open read fragment, ORF) of *wetA* (167 to 308 bp downstream of the TSS). (i) Comparation of the enriching levels of the H3K36me3-modified chromatin fragment of *steA* gene between WT and *δsetb/asha* strains. (j) the expression level of the sclerotia regulating gene *SteA*. (k) ChIP-qPCR analysis on H3K36me3 modified ORF region of *steA* gene (1909 to 2023 bp downstream of the TSS).

Catalysed by both Set2 and Ash1, H3K36me3 in *F. fujikuroi* is deeply involved in the development, mycotoxin synthesis and the formation of virulence of the fungus [29]. To the methylation of H3K36, only trimethylation is correlated with transcription rates [22,30,31]. The trimethylation of H3K36 in this study was abolished in the double deletion strain (*ΔsetB/ashA*). To discover the mechanism by which H3K36me3 regulates the aflatoxin synthesis and fungal virulence, ChIP-seq analysis was carried out with anti-H3K36me3 monoclonal antibody to enrich H3K36me3 modified chromatin fragments from WT strain using *ΔsetB/ashA* strain as a control. The heat map (Figure S7A) reflected that the quality fungal samples are competent for ChIP-seq analysis. The distribution analysis of the peaks enriched across the genome showed that the up enriched peaks from WT strain versus *ΔsetB/ashA* strain are distributed on the whole genome of *A. flavus* (Figure S7B). Most up-enriched peaks in WT are located in exon (46.88%) and promotor (41.25%) regions (Figure S7C), and most peaks are composed of promotor, exon and intron as shown in Figure S7D. All 5592 peaks were identified in the ChIP-seq analysis, among which 2018 peaks are differently accumulated (differently accumulated peaks, DAPs) in WT or Δ*setB/ashA* fungal strain. In these 2018 DAPs, 1995 DAPs are significantly accumulated in WT *A. flavus* strain (Red colour) and 23 DAPs are accumulated in the ΔsetB/ashA strain (green colour). And the left 3574 peaks are not differentially regulated (black colour) (Figure 4(a)). The significant peaks enriched in the WT strain (the peak value is at least twice that of the *ΔsetB/ashA* strain) are listed in Table S2. The Go clustering for up-enriched gene in WT strain (Figure 4(b)) showed that these up-enriched genes extensively involved in the regulation of various kind of biological processes (including cellular process, metabolic process, cellular component organization or biogenesis, and biological regulation) with different molecular functions (mainly including catalytic activity, binding, transporter activity, and transcription regulator activity). And in the bubble graph of KEGG classification (Figure 4(c)), the pathway of amylose and sucrose metabolism is the only one, in which the *p* volume is smaller than 0.5, which reflected that the pathway may play an important role in the secondary metabolism of this pathogenic fungus under trimethylation of H3K36. Above results confirmed that H3K36me3 is closely related to the regulation of various important biological processes, such as metabolic process, signalling, biological regulation, reproduction and growth revealed in this study.

### *AshA and SetB directly regulate fungal reproduction through trimethylation of H3K36 on the pathway regulator* wetA *and* steA

It is reported that the sporulation of filamentous fungi is regulated by AbaA, AblA and WetA, and WetA activates a series of genes involved in the spore wall impermeability and finally mature [32–34], so the normal expression of *wetA* gene is the last regulation step for sporulation. The peak map from ChIP-seq showed that, to *wetA* gene, the enrichment level of H3K36me3 modified promotor and ORF regions in WT strain is obviously higher than that in the *ΔsetB/ashA* strain (Figure 4(d)). Further qRT-PCR assay displayed that the transcriptional level of *wetA* gene in WT is remarkably higher compared to that in the *ΔsetB/ashA* strain (Figure 4(e)). To make it clear that if the activity of *wetA* was regulated by the trimethylation of H3K36, ChIP-qPCR was further carried out, which verified that the enrichment amount of H3K36me3 modified chromatin fragment, including the promotor and ORF regions of *wetA* gene in WT strain is extremely significantly higher than that in *ΔsetB/ashA* strain (Figure 4(f–h)). The transcriptional factor SteA (belonging to homeodomain-C2/H2-Zn + 2 finger) is indispensable for the sexual reproduction of *A. nidulans* [35]. The sexual reproductive structures of *A. flavus*, the ascospore-bearing ascocarps, are embedded within sclerotia [36]. According to the peak map, the ORF region of *steA* is significantly enriched in WT strain compared to *ΔsetB/ashA* strain (Figure 4(i)). The results from the qRT-PCR analysis in Figure 4(j) coincide the results from Figure 3(f). No sclerotia were found in *ΔsetB/ashA* strain (Figure 3(f)), and the expression level of the sclerotia formation regulator *steA* of *ΔsetB/ashA* strain was also significantly decreased (Figure 4(j)). The further ChIP-qPCR showed the ORF of *steA* in WT strain was enriched, and confirmed that the activity of *steA* was regulated by H3K36me3 (Figure 4(k)). Above results reflected that the Set2 family methyltransferases directly promote fungal asexual propagation via catalysing H3K36me3 in the promotor and ORF chromatin regions of *wetA*, and boost sexual reproduction via catalysing H3K36me3 in the *steA* ORF chromatin region.

### *H3k36me3 modification on* aflR *chromatin regions directly up-regulates AFB1 anabolism*

**Figure 5.**
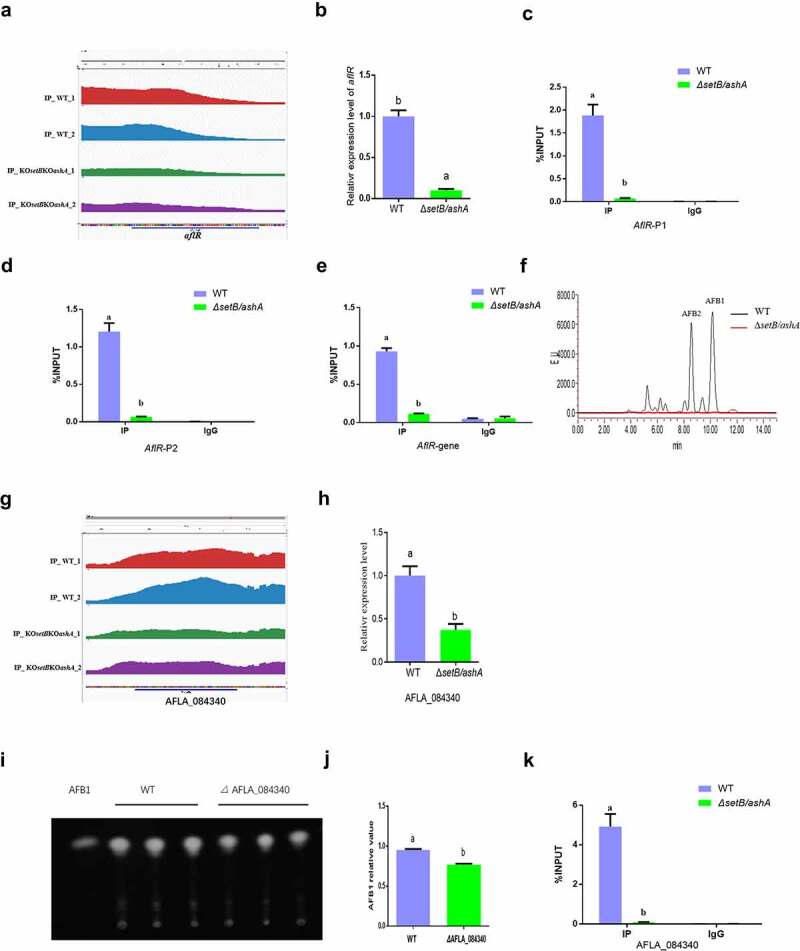
SetB and AshA regulate the biological synthesis of AFB1 through catalyzing the trimethylation of H3K36. (a) Comparation of the enrichment of H3K36me3-modified *aflR* chromatin fragments between WT and *δsetb/asha* strains (Red and blue peaks are the repetition for WT strain; green and purple are repetition for *δsetb/asha* strain). (b) qRT-PCR analysis monitoring the expression level of AFB1 biological synthesis regulating gene *aflR*. (c) ChIP-qPCR analysis on the promotor of regulating gene *aflR* (at the position of −627 to −491 bp upstream of the TSS, IgG was used as negative control). (d) ChIP-qPCR analysis on the second position of *aflR* promotor (−197 to −93 bp upstream of the TSS). (e) ChIP-qPCR analysis on the ORF region of *aflR* (337 to 520 bp downstream of the TSS). (f) HPLC analysis on the AFB1 production of WT and *δsetb/asha* strain (The black curve is for the WT strain, the red curve is for the *δsetb/asha* strain). (g) Comparation of the enrichment of H3K36me3-modified chromatin fragments of the amylase gene (AFLA_084340) between WT and *δsetb/asha* strains (Red and green peaks are the repetition for WT strain; blue and yellow are repetition for *δsetb/asha* strain). (h) qRT-PCR analysis monitoring the expression of the amylase gene. (i) TLC analysis of AFB1 produced by WT strain and the amylase gene knock-out strain (*Δ*AFLA_084340). (j) the histogram showing the relative production of AFB1 in panel I. (k) ChIP-qPCR analysis on the ORF of AFLA_084340 (at the position of 1609 to 1740 bp downstream of the TSS, IgG was used as negative control).

In the AFB1 synthesis cluster, the most important regulator for AFB1 biological synthesis is AflR. The peak map showed that the H3K36me3 levels of the promotor region and the gene coding area of the regulator in WT strain were remarkably higher compared to that in the *ΔsetB/ashA* strain (Figure 5(a)). Following qRT-PCR analysis displayed that the transcriptional level of *AflR* in WT strain is remarkably higher compared to that in the *ΔsetB/ashA* strain (Figure 5(b)). ChIP-qPCR analysis on the promotor regions confirmed that the H3K36me3 modified *aflR* promotor fragments in WT strain is significantly enriched compared to that in the *ΔsetB/ashA* strain (Figure 5(c,d)). Further ChIP-qPCR verified that *aflR* ORF region is significantly trimethylation-modified at H3K36 in WT compared to *ΔsetB/ashA* strain (Figure 5(e)). To reveal whether the activity of Set2 family affects the anabolism of AFB1, HPLC was further performed and the result revealed that the absence of H3K36me3 from *aflR* promotor and ORF regions in *ΔsetB/ashA* strain inhibits the production of AFB1 and AFB2 (Figure 5(f)), which reflected that the H3K36me3 level of the regulator gene regulates the anabolism of AFB1 in *A. flavus*.

### Set2 family methyltransferases promote mycotoxin anabolism via substrate availability

As the building block of all known fungal polyketides, Acetyl-CoA is generated by various biochemical pathways, including glycolysis of carbohydrates [37]. As a polysaccharide, starch is an important source of Acetyl-CoA. In our ChIP-seq assay, it was found that amylose and sucrose metabolism is the only pathway in which the *p*. adjust value is below 0.5 in the KEGG analysis (Figure 4(c)). And the peak map showed that the H3K36me3 level in the ORF area of the amylase gene (AFLA_084340) in the carbohydrates metabolism pathway in WT strain is significantly higher, compared to that of the *ΔsetB/ashA* strain (Figure 5(g)). To test if the amylase gene is regulated by Set2 family methyltransferases, qRT-PCR was performed, which reflected that the transcriptional level of the amylase gene in the *ΔsetB/ashA* strain was remarkably repressed, compared to that of the WT strain (Figure 5(h)), indicating that the amylase gene was regulated by the methyltransferase family. To test if the amylose participates in mycotoxin anabolism, the amylase gene deletion strain (*Δ*AFLA_084340) was constructed. The further TLC assay was performed, and the result showed that the absence of the amylase suppresses the production of AFB1 (Figure 5(i,j)). To verify if the amylase was regulated by H3K36me3, ChIP-qPCR was further performed, and the result showed that the H3K36me3 was significantly accumulated in the AFLA_084340 gene ORF chromatin region of the WT strain, compared to the *ΔsetB/ashA* strain (Figure 5(k)). Above results reflected that Set2 family methyltransferases up-regulated AFB1 anabolism via H3K36 trimethylation at substrate utilization level.

## Discussion

**Figure 6.**
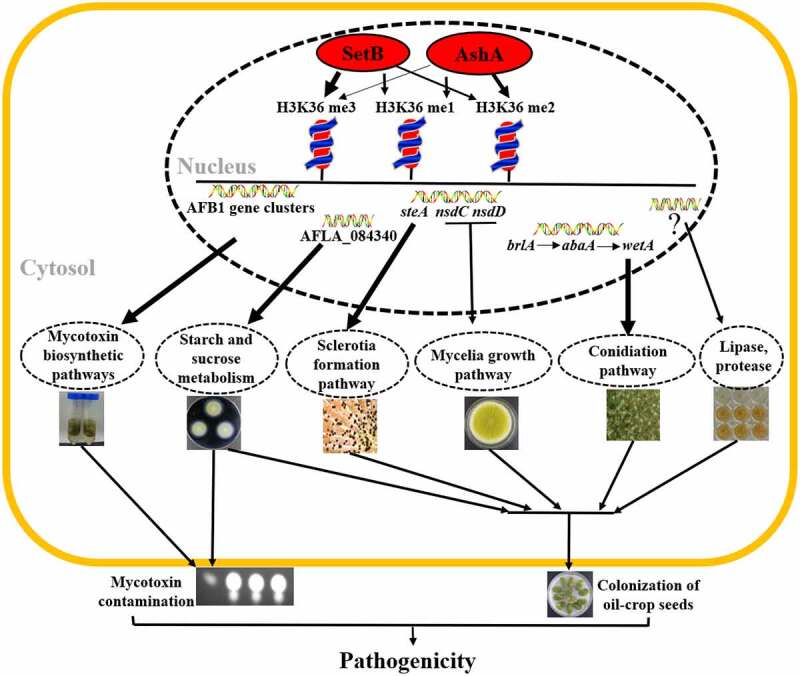
The regulatory model of AshA and SetB in the virulence of *A. flavus*.

Over the past decade, it has been found that epigenetic regulation plays a key role in tuning of eukaryotic gene expression. Epigenetic regulation refers to the heritable changes in chromatin conformation primarily by histone post-translational modification (HPTM). These modifications are important for the recruitment of other chromatin remodelling and transcriptional complexes [38]. Histone methylation catalysed by Ash1 and Set2 is found in vertebrates, insects, fungus, and plants [39–43]. In our study, high homologous AshA and SetB homologs were found among *Aspergillus spp* when amino acid sequences were aligned (Figure S1A and S1B; Figure S5A and S5B), which suggested that the AshA and SetB may play similar important roles in the biological processes of filamentous fungi. However, no study on AshA and SetB homologues in *Aspergillus spp*. is reported yet. Through deletion and complementation of the *ashA* homologue gene in the *A. ﬂavus*, it was found that AshA regulates the conidiation of *A. flavus* on YES medium through *brlA* and *abaA* (Figure 1(b,c)). Both BrlA and AbaA are transcription factors involving in the formation of conidiophore and phialide [32–34]. But in the process of *A. flavus* invading hosts, especially in the silkworm mode, the conidiation of *A. flavus* was obviously down-regulated when AshA was absent (Figure 2(c,f–g)), which is coinciding with the bio-function of Ash1 in mammals or insects to enhance the activity of target genes [23,24,44]. The reduction in conidiation in pathogenic *Aspergillus spp*. mutants on the host surface would obviously reduce the chance of these pathogenic fungi to spread among hosts. The study also found that the deletion of *setB* gene significantly reduces the hyphal growth on crop kernels, and dramatically suppresses the production of conidia on both medium and the surface of crop kernels through *abaA* and *brlA* regulated pathway (Figure 3(c–e,h,m,n)), which is similar to what was observed in *F. fujikuroi* [29]. As sexual reproductive structures, sclerotia are generally considered to be a kind of survival structure against adverse conditions, and both *nsdC*, *nsdD* and *steA* are required for the production of sclerotia [35,45]. The *nsdC* up-regulates vegetative growth in *A. nidulans*, and the absence of *nsdD* inhibits radial growth of *A. flavus* [45,46]. In this study, we found that the absence of AshA or SetB decreased sclerotia production through *nsdC*, *nsdD* and *steA* genes, and might decrease the hyphal growth rate through *nsdC* and *nsdD* (Figures 1(d–f) and 3(f,g,i)). All these results reflected that the Set2 family methyltransferases play critical role in the development and propagation of the pathogenic fungus (Figure 6).

By mainly catalysing the methylation of H3K36 (the thickness of these arrows from AshA and SetB to methylation1–3 in above figure means the participation level of these two histone methyltransferase), nucleus accumulated AshA and SetB increase mycotoxin anabolism at transcriptional level and substrate level (thicker arrow means that the results has been confirmed with ChIP-seq, qRT-PCR and ChIP-qPCR), improves the sclerotia formation (the result has been supported by ChIP-seq, qRT-PCR and ChIP-qPCR analysis) and mycelial growth, enhances conidiation (the result has been supported by ChIP-seq, qRT-PCR and ChIP-qPCR analysis), and promotes the activity of digestive enzymes (amylase has been supported by ChIP-seq, qRT-PCR and ChIP-qPCR analysis). With all of the means listed above, The Set2 family methyltransferases promote mycotoxin anabolism, and boost virulence of *A. flavus* to crops and animals.

In the YES liquid stationary cultures, crop infection model and silkworm model, it was found that AshA involves in the anabolism of AFB1. Our results also revealed that the biosynthesis level of AFB1 and AFB2 decreased in liquid stationary cultures by down-regulating the expression level of orthodox aflatoxin gene cluster in *ashA* deleted mutant strain (Figure 1(j,k)). The gene *aflR*, coding a 47 kDa zinc-finger protein, is indispensable for the transcription of most structural genes of the aflatoxin gene cluster [7]. The *aflS* gene, adjacent to *aflR*, is bidirectionally transcribed from *aflR*. When *aflS* gene was knocked out, the transcriptional levels of some aflatoxin pathway genes were found to be 5- to 20-fold reduction [7]. Therefore, the absence of AshA down-regulated the aflatoxin production by orthodox aflatoxin genes cluster through regulators AflR and AflS. We also found that SetB is deeply involved in the biological synthesis of AFB1 in liquid medium or on crop kernels through AflR regulated aflatoxin gene cluster (Figure 3(j–l,o,p)). All these results reflected that both SetB and AshA involve in the regulation of the aflatoxin anabolism through the orthodox aflatoxin gene cluster. Starch is the common raw material for conversion and anabolism of aflatoxins by amylase in *A. flavus* [47]. The study found that Set2 family methyltransferases up-regulate AFB1 anabolism not only by increasing the expression levels of the transcriptional regulator genes (including *aflR* and *aflS* in aflatoxin gene cluster), but also by promoting the expression level of the starch digestive enzyme gene, AFLA_084340 (Figure 5(g–k)). In conclusion, Set2 family methyltransferases comprehensively regulate aflatoxin anabolism, not only through the aflatoxin gene cluster level but also by substrate utilization level.

As an important PTM, the methylation of H3K36 is conservative in eukaryotic creatures, and it is deeply involved in the fungal virulence as found in *F. fujikuroi* [29,48]. To discover the role of AshA in H3K36 methylation, we identify all the possible sites which were methylated by AshA, including H3K4, H3K36 and H3K79. Our study revealed that AshA was only responsible for H3K36me2 in *A. flavus* (Figure 2(p)). Previous reports proposed that Ash1 is involved in the H3K36me2 in drosophila [39], but MoKMT2 H (Ash1-like protein), in *M. oryzae* doesn’t affect the modification of H3K36me2/3 [27]. The results of our study showed a distinct methylation spectrum of AshA in *A*. *flavus*, compared with other fungi, and it is more similar to the methylation function of Ash1 in drosophila. Different from the report that Set2 cooperates Ash1 to methylate H3K36me3 in *F. fujikuroi* [29], our western-blotting results showed that SetB is mainly responsible for H3K36me3, with some degree of functional redundancy, by which SetB can participate in part of H3K36me2, and fully catalyse H3K36me when AshA is absent (Figure 2). AshA mainly catalyses H3K36me2, and very limited participates in the H3K36me3 (Figure 2). The absence of SetB results in the heavy accumulating of H3K36me and H3K36me2 (Figure 3(a)), which reflected that SetB is critical in the transferring H3K36me and H3K36me2 to H3K36me3. It is also indicated that all levels of methylation for H3K36 are fulfilled by AshA and SetB in filamentous fungi, because the double deletion of *ashA* and *setB* genes abolish all signals of H3K36 methylation (from monomethylation to trimethylation), and that is consistent with what discovered in H3K36A strain (Figure 3(a)).

SET domain is assembled by small β sheets enclosing a knot-like structure, in which an active site was formed just next to the peptide-binding cleft and to the pocket where the methyl donor binds, and mutation of SET domain impacts the enzymatic activity of SETD2 [49,50]. Our study showed that SET domain deeply participated in fungal development, mycotoxin anabolism, and the formation of virulence in *A. flavus* (Figures 3, S3 and S4). We also found that the mutation of N455 in *ashA*^N455A^ mutant and V457 in *ashA*^V457D^ mutant prevented the formation of sclerotia, suppressed the production of mycotoxins, and decrease the virulence of *A. flavus* (Figures S3 and S4). These results suggested that the polar state of N455 and V457 in SET domain is important for the activity of the Set2 methyltransferase family. All together showed that SET domain, and N455 and V457 in the SET domain are very important for the bio-function of SetB and AshA in *A. flavus*.

This study also explored the co-operational roles of AshA and SetB, and the results showed that compared to other *A. flavus* strains, including *ΔashA* and *Δ*setB mutants, the absence of both AshA and SetB significantly suppressed the vegetative growth, sclerotia formation, AFB1 synthesis, and fungal virulence of *A. flavus*. H3K36A mutant was prepared to reveal the biological function of H3K36 methylation in the virulence of this pathogenic fungus. And the most interesting is that, like point mutant fungal strain H3K36A, the methylation of H3K36 of *ΔsetB/ashA* are totally abolished as shown in Figure 3(a), which infers that all methylation levels of H3K36 in *A. flavus* are solely implemented by both AshA and SetB. And the comparation of the phenotypes of *ΔsetB/ashA* and H3K36A *A. flavus* strains in hyphal growth, sporulation, sclerotia formation, toxin anabolism and virulence to crops supported our above point of view, i.e. AshA and SetB regulate the virulence of the fungus by the methylation of H3K36.

This study reveals that both AshA mediated H3K36me2 and SetB mediated H3K36me3 are very important in the development and virulence of *A. flavus*. As shown in the results of the study, the lack of H3K36me2 in Δ*ashA* leads to several suppress against sclerotia formation, AFB1 production and the virulence of *A. flavus* to crops. In *S. cerevisiae* Set2p mediates H3K36me2, and the modification of H3K36me2 within ORFs correlates with the active or inactive state of transcription [31]. The lack of H3K36me3 in Δ*setB* in this study also results in significant decrease of conidiation, sclerotia formation, AFB1 anabolism, and the virulence of the fungus. In *F. fujikuroi*, H3K36me3, catalysed by Set2 and Ash1, plays a critical role in the development, secondary metabolism and fungal virulence [29]. To H3K36 methylation, among mono, di and trimethylation, mainly trimethylation displays positive correlation with the transcription of genes [22]. To reveal the modification mechanism of H3K36me3 in *A. flavus*, ChIP-seq assay was carried out. In ChIP-seq analysis, it was found that the epigenetic marker of H3K36me3 is distributed in the whole genome of *A. flavus*. The H3K36me3 modification is found to be mainly enriched on the exon and promotor regions of target genes (Figure S7). Among 5592 peaks, 2018 DAPs were identified, and most of the DAFs (1995 DAPs) were accumulated in the WT strain (Figure 4(a)). The KEGG analysis and Go annotation showed that, in many cellular components, the methylation of H3K36 plays essential roles in controlling and regulating various kinds of biological processes and different molecular functions (Figure 4). According to the H3K36me3 modified chromatin fragment accumulation peak map, three important regulator genes (*wetA* for asexual reproduction, *steA* for sexual reproduction and *aflR* for aflatoxin biological synthesis) and one amylase gene (AFLA_084340) were screened out for further analysis, and qRT-PCR showed the normal modification level of H3K36me3 is critical for maintaining the functional expression levels of both asexual and sexual production, and AFB1 synthesis related regulators and substrates. Further ChIP-qPCR analysis confirmed that *wetA* and *aflR* are trimethylation modified on both promotor and ORF chromatin sequences, and that *steA* and AFLA_084340 are modified on the ORF chromatin sequence (Figures 4 and 5). The work discovers that the trimethylation of H3K36 is indispensable in the biological processes of asexual and sexual reproduction and aflatoxin synthesis, which also suggested that the H3K36me3 modification plays a critical role in fungal development, mycotoxin anabolism and virulence of pathogenic filamentous fungi.

Overall, we explored and clarified bio-function of the nucleus located Set2 family methyltransferases in *A. ﬂavus*, and determine the influence of histone methylation mediated by both AshA and SetB on mycotoxin anabolism and fungal virulence to plant and animal (Figure 6). This study revealed that the AshA and SetB are responsible for all levels of H3K36 methylation in *A. flavus*, through which they directly regulate mycotoxin synthesis and fungal virulence of the pathogenic fungus. Our study revealed the potential epigenetic machinery regulating the morphogenesis, mycotoxin anabolism and fungal virulence attributes of *A. flavus*, and provided a novel perspective for developing new prevention and control strategies against pathogenic fungi.

## Materials and methods

### Ethics statement

The animal related experiments in the study were performed strictly following the animal welfare guidelines set by the World Organization for Animal Health, and approved by the institutional ethics committee of the Fujian KLPFM (Permit number: PFMFAFU201680).

### Fungal strains

The *A. flavus* strains constructed and utilized in the study were listed in Table 1. Media used in this study were YPD (2% peptone, 1% yeast extract, and 2% glucose, and 1.5% agar), CM (0.6% tryptone, 0.6% yeast extract, 1% sucrose), and YES (0.5% yeast extract, 2% glucose, and 1 mL/L trace elements prepared as introduced) [45]. Finally, 1.5% agar was added for solid media, and both uridine and uracil were provided as required for the auxotrophic marker, *pyrG*- [45]. All the strains were kept in 30% glycerol at −80°C.

**Table 1.** *A. flavus* strains used in this study.

Strain name	Related genotype	Source
PTSΔ*ku70*Δ*pyrG*	Wild type (*pyrG-*)	Kindly presented by Prof. Chang [51]
PTSΔ*ku70*	Wild type (*pyrG+*)	Kindly presented by Prof. Chang [51]
Δ*ashA*	Δ*ku70*, Δ*ashA::pyrG*	This study
*ashA*^*ΔSET*^	Δ*ku70*, *ashA*^*ΔSET*^::*pyrG*	This study
*ashA*^N455A^	Δ*ku70*, *ashA*^N455A^::*pyrG*	This study
*ashA*^V457D^	Δ*ku70*, *ashA*^V457D^::*pyrG*	This study
Com-*ashA*	Δ*ku70*, Δ*ashA:pyrG*, *ptr-ashA*	This study
*mCherry-ashA*	Δ*ku70*, *pyrG-gpda(p)-mCherry-ashA*	This study
*ΔsetB*	Δ*ku70*, Δ*setB::pyrG*	This study
Com*-setB*	Δ*ku70*, Δ*pyrG*; Δ*setB::pyrG::setB*	This study
setB^*ΔSET*^	Δ*ku70*, *setB*^*ΔSET*^::*pyrG*	This study
*ΔsetB/ashA*	Δ*ku70*, Δ*setB:pyrG*, Δ*ashA:ptr*	This study
H3K36A	Δ*ku70*, *H3*^K36A^::*pyrG*	This study
*setB-mCherry*	Δ*ku70*, *setB-mCherry-pyrG*	This study

Com, means complementation.

### Bioinformatics analysis

AshA homologs from 16 fungal species (including *A. flavus* (×P_002384179.1), *A. oryzae*, *A. parasiticus*, *R. emersonii*, *A. nomius*, *A. kawachii*, *A. niger*, *A. clavatus*, *A. luchuensis, A. fischeri*, *A. terreus*, *A. lentulus*, *A. udagawae*, *A. fumigatus*, *T. marneffei*, and *T. islandicus*) were downloaded from the NCBI blast (The website: http://www.ncbi.nlm.nih.gov). Phylogenetic tree of the AshA protein of all these 16 species was further established with the software MEGA5.1 with the algorithm of Neighbouring comparison (1000 times). Protein domains of these 16 species was analysed with the software SMART (The website: http://smart.embl-heidelberg.de/) and edited with IBS 1.0. The homologs of SetB from the above strains were also analysed following the methods mentioned above.

### *Generation of* Aspergillus flavus *mutants*

The *ashA* deletion and complementation were constructed as previously described by Han (2016) with minor modification [52]. 5’- and 3’-untranslated regions (about 1.2 kb respectively) were amplified with primers *ashA*-p1 to *ashA*-p4 (Table S1) from *A. flavus* genomic DNA. As the selecting marker, the *pyrG* gene was amplified with primers *ashA*-p5 and *ashA*-p6 (Table S1). Followed by the three fragments, including both 5’-UTR and 3’-UTR fragments of *ashA*, and the *pyrG* gene fragment, were ligated together via fusion PCR with a pair of nesting primers *ashA*-p7 and *ashA*-p8 (Table S1). To the *pyrG* prototroph *A. flavus* strain (Δ*ashA*: Δ*ku70*, Δ*ashA::pyrG*, refer to Table 1), the whole *ashA* coding sequence was replaced by *pyrG*, and the *ashA* gene knockout fungal strain Δ*ashA* was further verified by Southern-blotting and PCR (The related primers are listed in Table S1) [53]. The *ashA* gene complementation fungal strain (Com-*ashA*) was prepared through transforming the *ashA* deletion mutant Δ*ashA* with the *ashA* wild type allele with *ptrA* as selection marker, and a 1030 bp fragment of *ashA* ORF was amplified with primer *ashA*-p10 and *ashA*-p11 to confirm that *ashA* was induced back into Δ*ashA* strain. SET domain deletion mutant (*ashA*^*ΔSET*^) and point mutant strains (*ashA*^N455A^ and *ashA*^V457D^) were generated according to the method mentioned above, and the primers were listed in Table S1. The mutated amino acids (Asparagine 455 and Valine 457) of the SET domain sequence in *A. flavus* were located by MEME at http://meme-suite.org. The mutants were finally confirmed through sequencing by BioSune Biotechnology (Shanghai) Co., Ltd.

To construct *setB* gene deletion strain (Δ*setB*), 5’UTR (amplified with *setB*-p1 and *setB*-p2), 3’UTR (amplified with *setB*-p3 and *setB*-p4) and *pyrG* (amplified with *setB*-p5 and *setB*-p6) were ligated together with a pair of nesting primer *setB*-p7 and *setB*-p8. The Δ*setB* strain was constructed by transforming the protoplast (PTSΔ*ku70*Δ*pyrG*) with the fusing production, and tested by diagnostic PCR and southern-blotting analysis. The construction of *setB* complementary strain was following the protocol provided by Hu [54], in which the *pyrG* in Δ*setB* was replaced by 5’-UTR-*setB*-3’-UTR (amplified by primer *setB*- p1 and *setB*- p4; template: G-DNA) under 2 mg/mL 5-fluoroorotic acid (5-FOA), and *pyrG* gene was fused with 5’UTR (amplified with primer *setB-C*-p1 and *setB-C*-p2) and 3’UTR (amplified with primer *setB-C*-p3 and *setB-C*-p4) with nesting primer *setB-C*-p7 and *setB-C*-p8, and *pyrG* gene was inserted into the end of *setB* gene of *setB* complementary strain by homologous recombination as shown in Figure S5E. The final complementary strains were further identified through q-RCR and diagnostic PCR. NCBI database analysis revealed that the 302–530 amino acids in the protein sequence of SetB are the SET Domain amino acid sequence, and the SET domain deletion fungal mutant *setB*^*ΔSET*^ was prepared by the homologous recombination method used above.

### Quantitative reverse transcription-polymerase chain reaction (qRT-PCR) assay

The qRT-PCR assay was performed following the former method described by Zhang (2016) [55]. To remove possible residual G-DNA, the total RNA (5 µg) was firstly treated with DNase I (Thermo Fisher Scientiﬁc, Waltham, MA, USA). Then, the G-DNA free RNA (1 mg) was reverse-transcribed into cDNA by using the Revert Aid First-strand cDNA Synthesis kit (Thermo Fisher Scientiﬁc, Waltham, MA, USA). Finally, the qRT-PCR was performed with the SYBR Green Premix kit (Takara, Dalian, China), and the instrument used is M×3000p thermocycler (Agilent Technologies). The method of 2´∆∆Ct was applied to evaluate the expression levels of corresponding target genes. The primers of the qRT-PCR assay were listed in Table S1.

### Morphological analysis

To estimate the development state of the fungal colony, related fungal strains were point-inoculated on corresponding media in dark at 37°C, and the diameters of fungal colonies were measured after 4 d (6 d in the dark for sclerotia observation). Seven-mm-diameter cores were collected from the location one centimetre away from colony centre, and homogenized in 500 μL water, and then the conidia number was counted by haemocytometer. The role of *ashA* in sclerotial formation was estimated according to the protocol described by Zhuang (2016) [56].

### Secondary metabolism assay

The assay of the role of SetB and AshA in AFB1 yield was carried out with TLC and HPLC analysis following the methods performed by Lan *et al* [57].

### Plant invasion analysis

Crop kernels, including corns and peanuts, were selected to assess the biological function of SetB and AshA in the infection of plant by *A. flavus* as provided by Yang (2016) with minor modifications [58].

### Animal invasion assay

Silkworms (*Bombyx mori*) were maintained in clean boxes with adequate tender mulberry leaves at about 28°C according to national animal care guidelines. When each silkworm larva reaches about 1 g in weight and 4 cm in length, the 4th-instar silkworms were separated into four groups (10 larvae/group), then, injected at the end of dorsal surface with 5 µL saline (set as the control group), and 5 µL conidial suspension (10^6^ spores/mL) from WT, Δ*ashA* and Com-*ashA A. flavus* strains. Dead silkworms from WT, Δ*ashA* and Com-*ashA* groups were transferred into 9 cm-Petri dishes (6 silkworm/dish), and inoculated for 6 d in the dark, and AFB1 from *A. flavus* contaminated silkworms was extracted with chloroform.

### Digestive enzyme activity assay

The lipase and amylase activity assay were carried out following the protocols provided by Li (2017) [59]. The assessment of the role of AshA in protease activity was implemented according to the methods used by Zhuang [56].

### Western-Blotting assay

The mycelia were inoculated on YES agar for 2 d under 29°C in the dark and lyophilized for 10 h. The mycelia were fully ground into a powder with steel beads, mixed with ice cold RIPA buﬀer (Beyotime, China) with proteinase inhibitor PMSF (1/1000 v/v) for 3 h, and treated by ultrasonication (40 KHz) for 3 m every 1 h on ice. The total lysate was analysed by the system of NuPAGE Novex Tris-acetate electrophoresis (Life Technologies, USA), about 60 μg of protein sample is loaded in each lane. Electrophoresis was carried out under the condition of constant voltage of 80 V for 30 min. Then the voltage was adjusted to 120 V, electrophoresis was continued until the protein maker bands were completely separated. Followed by the protein samples were shifted onto a PVDF membrane (0.25 μm) at 200 mA constant current for 30 min. Then, the protein coated membrane was immersed in TBST buffer (2.42 g Tris, 8 g NaCl chloride in distilled water, fix the volume to 1000 mL, and adjust the pH value to 7.6 with HCl, 1 mL Tween 20 was added finally) with 5% fat free milk overnight for sealing. The sealed membrane was incubated evenly with rabbit anti-H3K36me antibody (Cat#PTM-623, Lot#21450310P4), anti-H3K36me2 antibody (Cat#PTM-624, Lot#2146G031P3), anti-H3K36me3 antibody (Cat#PTM-625, Lot#2147D415P2) against target histone methylation site for 1 h at RT, at a dilution of 1:1000. Then incubated with horseradish peroxidase conjugated secondary antibody (goat anti-rabbit lgG, at a dilution of 1:5000) after extensive washing, and the reagent of ECL was applied for finally detection with G: BOX Chemi XT4 (Syngene, UK). H3 protein was detected as inner reference, relative methylation level calculated based on signal H3 [60].

### Subcellular location with mCherry

To localize AshA, a fusion PCR product with 1200-bp 5’-UTR of *ashA* (with *ashA*-p1 and 5-*pyrG*), 1890-bp *pyrG* (with *pyrG*-F1 and *pyrG*-R1 primers), 1510-bp *gpdA* promotor (with *gpdA*-F and *gpdA*-R primers), 710-bp *mCherry* gene (with mC-F and mC-R primers), and a 1040 bp fragment of *ashA* ORF (from the initiation codon, amplified with *ashA*-mC-F and *ashA*-mC-R primers) was amplified with *N-ashA*-m-F and *N-ashA*-m-R primers. The product of fusion PCR was used to transform the PTS Δ*ku70* (*pyrG*-) strain. The construction of SetB-mCherry co-expression fungal strain followed the method used above, and the related primers were recorded in Table S1. For SetB subcellular localization, a fusion PCR product with 1061 bp fragment of *setB* (with primer: *setB*-mCh-p1 and *setB*-mCh-p2), 710 bp *mCherry* gene (with *mCh*-F and *mCh*-R primers), 1890 bp *pyrG* (with *setB-mCh-pyrG*-F and *setB-mCh-pyrG*-R primers) and a 1155 bp 3’UTR (with *setB-mCh*-p3 and *setB-mCh*-p4 primers) was amplified with *setB-mCh*-p7 and *setB-mCh*-p8 primers. The product of fusion PCR was applied to transform the PTS Δku70 (*pyrG*-) strain. The transformants (*setB-mCherry* and *mCherry-ashA*) were PCR verified before used for *setB* and *ashA* location assay by confocal scanning laser microscopy.

### ChIP-Seq and ChIP-qPCR analysis

ChIP (Chromatin immunoprecipitation) was carried out following the protocol provided by Gendrel *et al*. (2005)[61] with some necessary modification. Conidia (10^4^/mL) of WT and *ΔsetB/ashA* strains were incubated in YES media at 29°C for 3 d. After hyphae was collected and mixed with 1% formaldehyde under vacuo for about 10 min, 2.5 mL of glycine solution (2 M) was added to stop the cross-link reaction under vacuo for 5 min. ChIP assays and ChIP-qPCR analysis were carried out following the previously provided protocol [61] by Wuhan IGENEBOOK Biotechnology Co.,Ltd (http://www.igenebook.com). (The primers applied in ChIP-qPCR were recorded in Table S1).

### Statistical analysis

In this study, the data were displayed with the means±standard deviation. Whether there was a statistically significant difference was determined by the one-way ANOVA, and when *P* < 0.05, the difference was statistically significant.

## Supplementary Material

Supplemental Material

## Data Availability

All the raw data of the ChIP-seq was uploaded to the SRA database with the accession number: PRJNA795870.
