# Supplementary material for: Set2 family regulates mycotoxin metabolism and virulence via H3K36 methylation in pathogenic fungus Aspergillus flavus
Source: Virulence. 2022 Aug 9;13(1):1358–78. doi: 10.1080/21505594.2022.2101218 (PMC9364737; doi:10.1080/21505594.2022.2101218)
Supplement: Supplemental Material [file KVIR_A_2101218_SM8872.zip › supplementary/Supplementary figure 2022-5-20.docx]

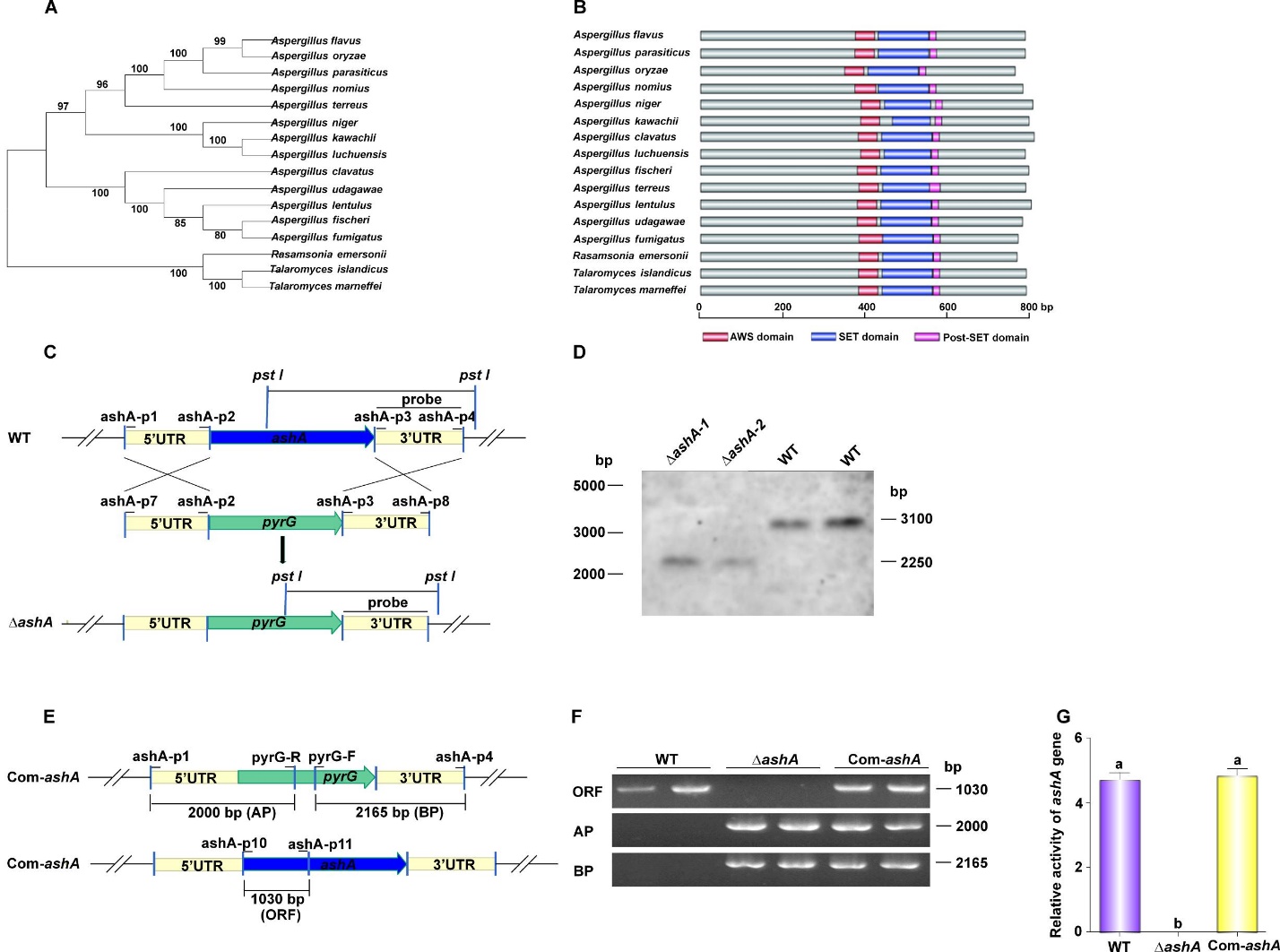


**Figure S1. Bioinformatics analysis, and the deletion and complementation of *ashA*.**

**(A**) Diagram showing the phylogenetic tree according to AshA sequences among 16 species: *A. flavus*, *A. parasiticus*, *A. oryzae*, *A. nomius*, *A. niger*, *A. kawachii*, *A. clavatus, A. luchuensis, A. fischeri*, *A. terreus*, *A. lentulus*, *A. udagawae*, *A. fumigatus*, *R. emersonii*, *T. islandicus* and *T. marneffei*. MEGA5.1 (with an algorithm of 1000 times Neighbering comparison) was used in the analysis.

**(B**) Diagram shows the domains in AshA among above 16 species. SMART (<http://smart.embl-heidelberg.de/>) and IBS 1.0 were used in the analysis.

**(C**) The scheme for Δ*ashA* construction by homologous recombination. 1.2 kb 5’-UTR and 1.2 kb 3’-UTR of *ashA* and 1.89 kb *pyrG* from *A. fumigatus* were amplified, and they were fused together with nesting primers *ashA*-p7 and *ashA*-p8 (**Table S1**). The *ashA* deletion strain was prepared with *pyrG* to replace *ashA* in WT (PTSΔ*ku70*Δ*pyrG*) by homologous recombination, and the DNA was digested by *pst* I and detected with the 3’-UTR probe for southern blot analysis.^[30]^

**(D**) The results of southern blot analysis. A band of 2250 bp was detected in Δ*ashA* strains and a 3100 bp fragment was detected from WT, which confirmed that *ashA* has been knocked out successfully.

(**E**) The diagram for the construction of Com-*ashA* strain.

(**F)** PCR analysis of Δ*ashA* and Com-*ashA* strain with primers showed on **A**. With Com-*ashA* G-DNA as template, a 1030 bp fragment from *ashA* ORF was amplified, and 2000 bp AP (from *ashA*-5’UTR and *pyrG*) and 2165 bp BP (from *pyrG* and *ashA*- 3’UTR) fragments were amplified.

(**G**) qRT-PCR result of the expression level of *ashA* in WT, *ΔashA* and Com-*ashA* strains, and *tubulin* was chosen as control (all primers were listed in **Table S1**).

The error bars represent the standard error. Different letters (“a” and “b”) above the bars represent significant different values (*p* < 0.05).

**
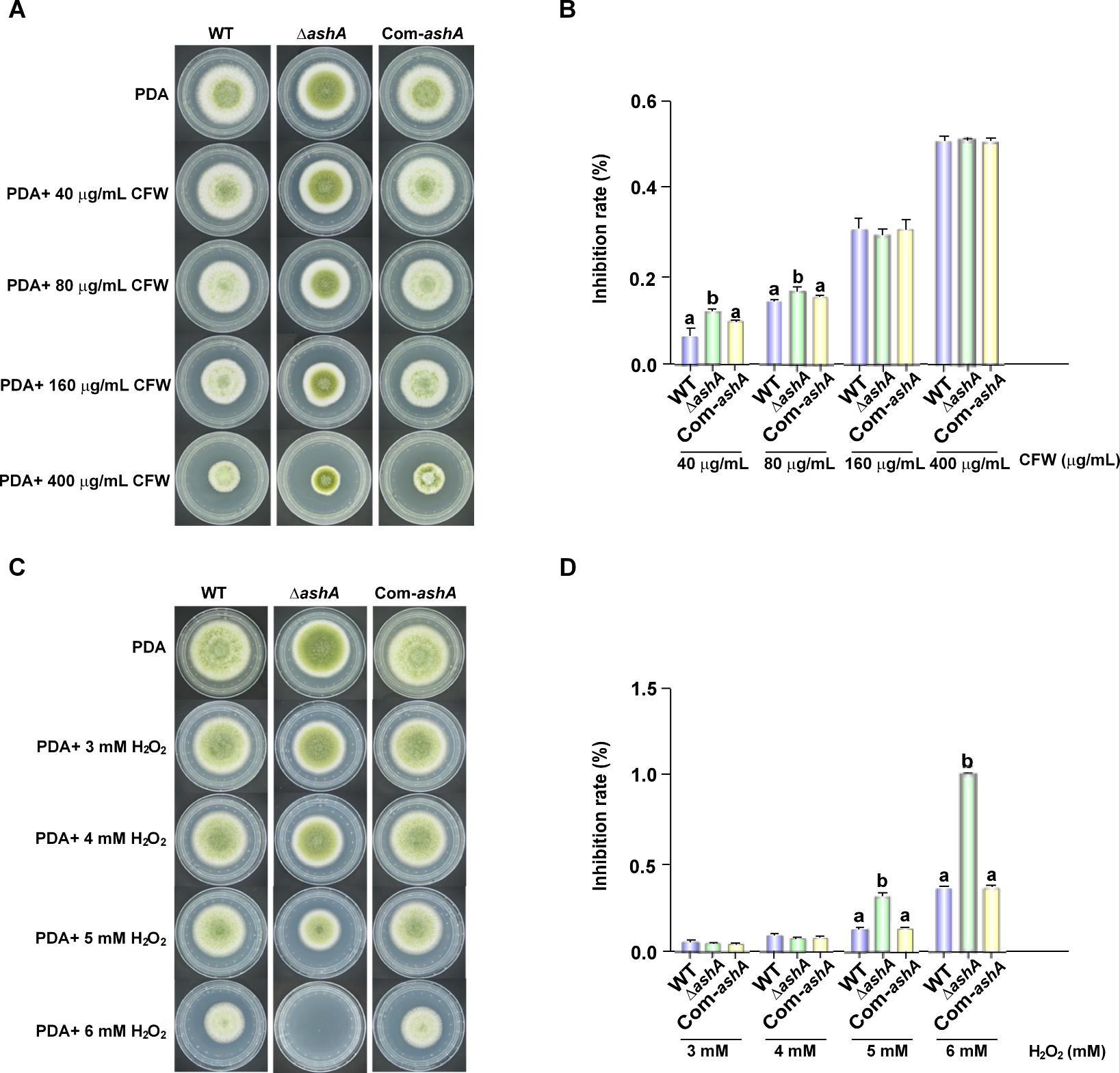
**

**Figure S2. The role of AshA in the sensitivity reaction of *A. flavus* to CFW and H_2_O_2_.**

(A) The *A. flavus* strains were inoculated on PDA media with a series of concentrations of CFW under 37℃ for 4 d.

(B) The histogram showing the inhibition rate of CFW to above fungal strains according to the results from the panel A.

(C) The fungal strains were inoculated on PDA media with a series of concentrations of H_2_O_2_ at 37℃ for 4 d.

(D) The inhibition rate of H_2_O_2_ to above *A. flavus* strains calculated according to the results of panel C.

Relative inhibition rate = (diameter of the colony without inhibitor - diameter of the colony with inhibitor) / diameter of the colony without inhibitor. The error bars represent the standard error (*P* < 0.05).


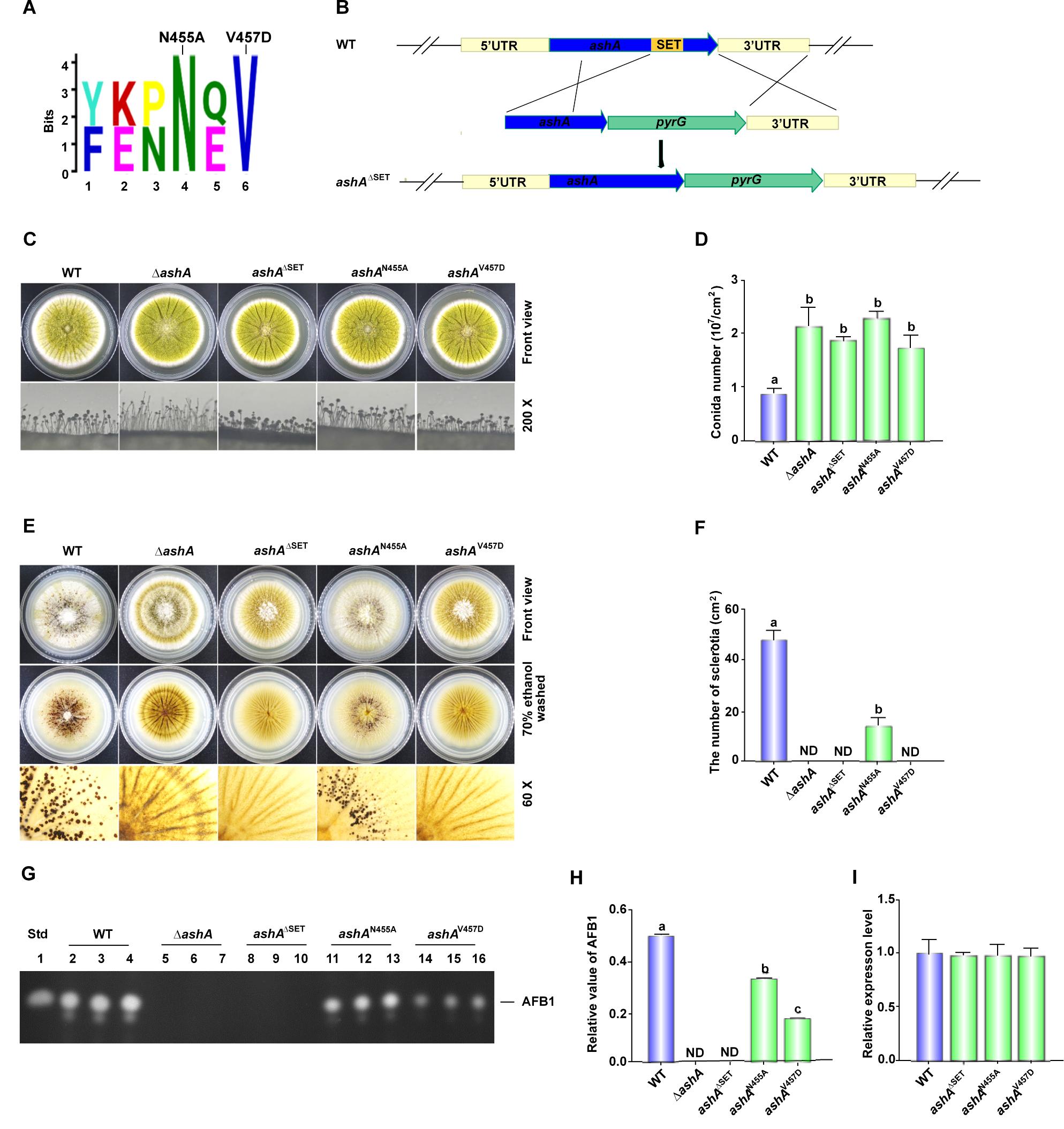


**Figure S3. SET domain, N455 and V457 of AshA are involved in the development and secondary metabolism of *A. flavus*.**

**(A**) The conserved motif with six amino acids was identified in *A. flavus* with MEME (<http://meme-suite.org>.), and the N455 and V457 were the most conserved amino acids among them.

(**B**) The scheme diagram showing the strategy for *ashA^ΔSET^* strain preparation.

(**C)** SET domain, N455 and V457 play an important role in the conidiation of *A. flavus*. The fungal strains were cultivated in YES medium at 37℃.

(**D**) Conidial quantification of the cultures of WT, Δ*ashA*, *ashA*^ΔSET^, *ashA*^N455A^, and *ashA*^V457D^ strains according to the results of **C**.

(**E)** SET domain, N455 and V457 are indispensable for the proper formation of sclerotia. The *A. flavus* strains were inoculated on CM media at 37^o^C for 6 d.

(**F**) Sclerotia quantification of the cultures of WT, Δ*ashA*, *ashA*^ΔSET^, *ashA*^N455A^, and *ashA*^V457D^ strains according to the results of **E**.

**(G**) TLC analysis of aflatoxin B1 production from WT, Δ*ashA*, *ashA*^ΔSET^, *ashA*^N455A^, *ashA*^V457D^, and Com-*ashA* strains after 6 d of incubation in liquid YES.

(**H**) Quantification of AFB1 production of *A. flavus* mutants listed above according to the result shown in **G**.

(**I**) The qRT-PCR analysis on the relative expression levels of *ashA* gene in the fungal strains of WT, *ashA*^ΔSET^, *ashA*^N455A^, *ashA*^V457D^. The above fungal strains were cultivated in liquid YES medium at 29℃ for 2 d.

The error bars represent the standard error (*P* < 0.05).

**
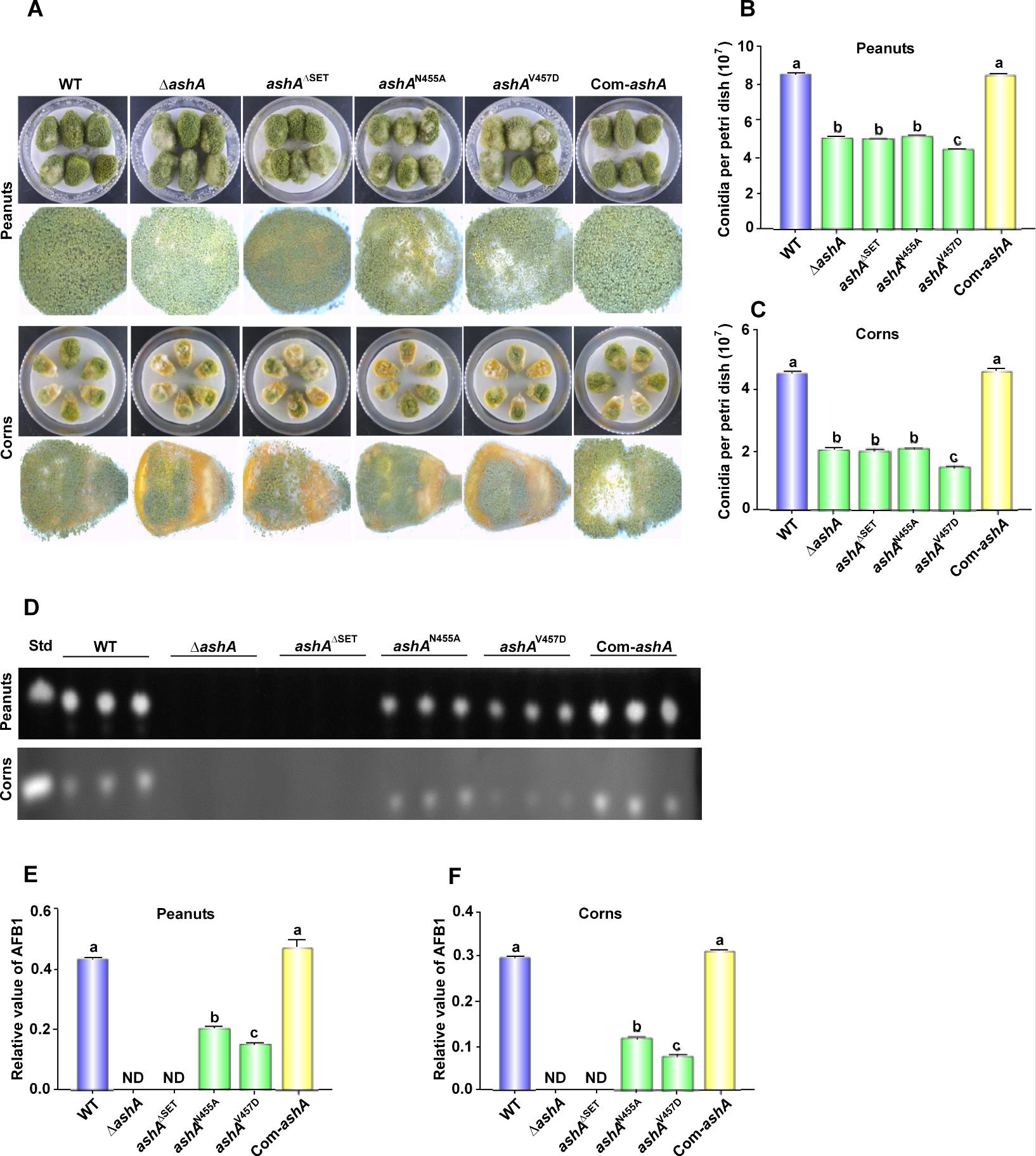
**

**Figure S4. SET domain, N455 and V457 are involved in the virulence of *A. flavus* to crop kernels.**

**(A**) The whole living peanut and corn kernels were incubated for a week at 28^o^C in the dark after mixed with 20 mL spores (10^5^/mL).

(**B**) The conidia number of each *A. flavus* strain on peanuts surface was calculated with hemocytometer.

(**C**) Histogram showing the number of conidia produced by each *A. flavus* strain on corn kernels.

(**D**) TLC analysis of AFB1 value produced by *A. flavus* colonized peanut and corn kernels.

(**E**) Relative amount of AFB1 in peanut seeds according to the results of **D**.

(**F**) Relative value of AFB1 in corn kernels according to the results of **D**.

The error bars represent the standard error (*P* < 0.05).


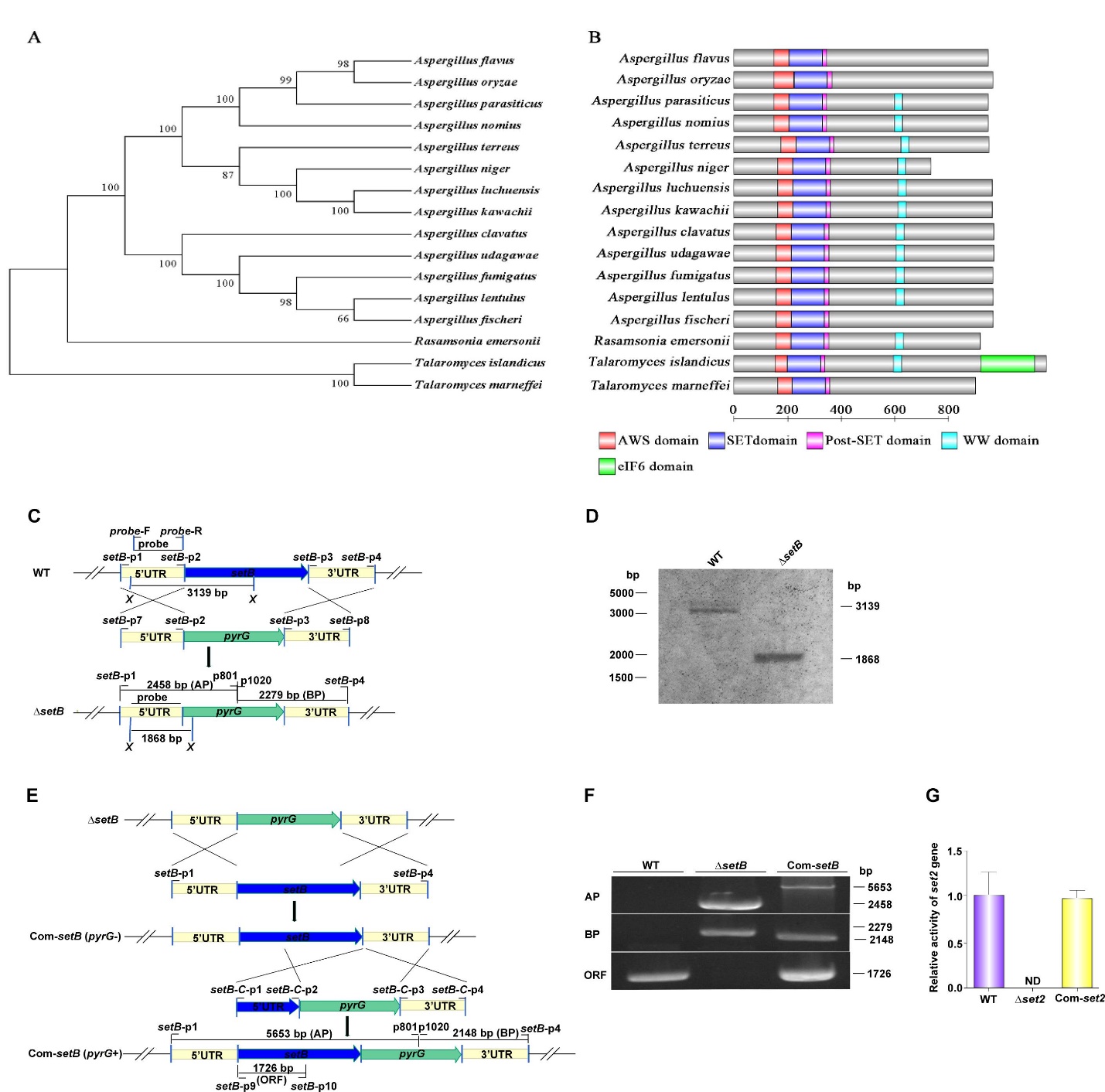


**Figure S5. Bioinformatics analysis, and deletion and complementation of *setB*.**

**(A**) Diagram showing the phylogenetic tree according to SetB sequences among16 species: *A. flavus*, *A. parasiticus*, *A. oryzae*, *A. nomius*, *A. niger*, *A. kawachii*, *A. clavatus, A. luchuensis, A. fischeri*, *A. terreus*, *A. lentulus*, *A. udagawae*, *A. fumigatus*, *R. emersonii*, *T. islandicus* and *T. marneffei*. MEGA5.1 was used in the analysis.

(**B**) Diagram shows the domains in SetB among above the 16 species. SMART (http://smart.embl-heidelberg.de/) and IBS 1.0 were used in the analysis.

(**C**) The scheme for Δ*setB* strain construction by homologous recombination. 5’-UTR (with primer *setB*-p1 and *setB*-p2) and 3’-UTR (with primer *setB*-p3 and *setB*-p4) of *setB* and 1.89 kb *pyrG* (with primer *setB*-p5 and *setB*-p6) from *A. fumigatus* were amplified, and they were fused together with nesting primers: *setB*-p7 and *setB*-p8. The *setB* deletion strain was prepared with *pyrG* to replace *setB* in WT (PTSΔ*ku70*Δ*pyrG*) through transformation the protoplast with the fusion production of 5’-UTR-*pyrG*-3’-UTR by homologous recombination, and the DNA was digested by *Xhol* I (*X*) and detected with the probe amplified with primer *probe*-F and *probe*-R for southern blot analysis.

**(D**) The results of southern blot analysis. A band of 1868 bp was detected in Δ*setB* strain and a 3139 bp fragment was detected from WT which confirmed that *setB* has been knocked out successfully.

(**E**) The diagram for the two-step construction of Com-*setB* strain according to the method used by Hu and her colleagues (2018).^[31]^

(**F**) The diagnostic PCR analysis of Δ*setB* and Com-*setB* strains with primers showed on **C** and **E**. With Com-*setB* G-DNA as template, a 1726 bp fragment from *setB* ORF was amplified with primer *setB*-p9 and -p10, 5653 bp AP fragment with primer *setB*-p9 and p801, and 2148 bp BP fragment with primer p1020 and *setB*-p4.

(**G**) qRT-PCR result of the expression level of *setB* in WT, *ΔsetB* and Com-*setB* strains. The qRT-PCR was performed using C-DNA from three *A. flavus* strains (WT, Δ*setB*, and Com-*setB*) as template with primers *Q-setB-F* and -*R* (**Table S1**), and tubulin (primers: Tubulin-F and Tubulin- R, **Table S1**) was chosen as a control. The result showed that the transcription level of *setB* was not detectable in Δ*setB* strain, but recovered in Com-*setB* strain. The error bars in **G** represents the standard error.


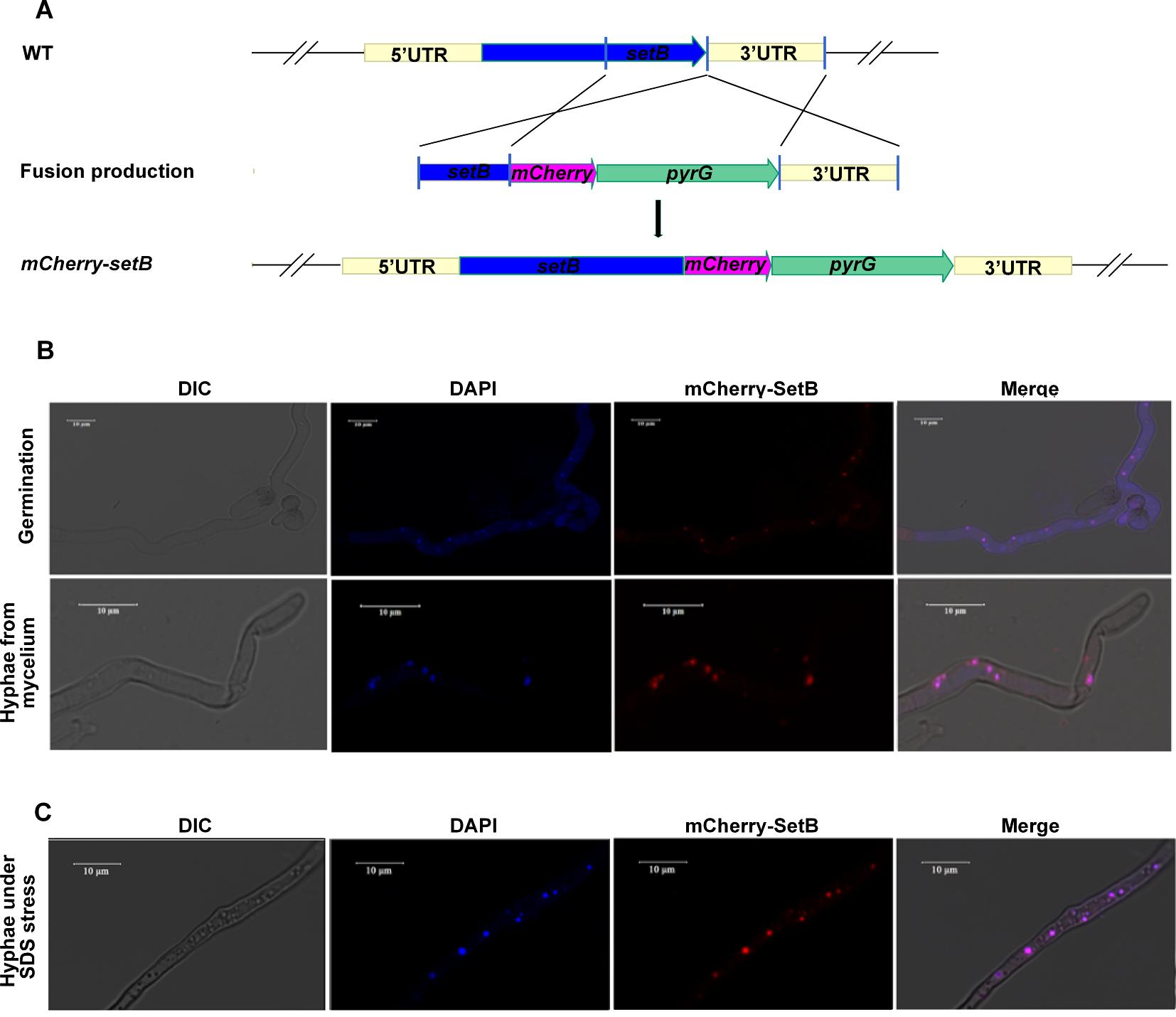


**Figure S6. The subcellular location of SetB.**

**(A**) The scheme for *setB-mCherry* strain construction by homologous recombination. The construction of SetB-mCherry co-expression fungal strain followed the method used above, and the related primers were listed in **Table S1**. For SetB localization, a fusion PCR product with 1061 bp fragment of *setB* (with primer: *setB*-mCh-p1 and *setB*-mCh-p2), 710 bp *mCherry* gene (with *mCh*-F and *mCh*-R primers), 1890 bp *pyrG* (with *setB*-*mCh*-*pyrG*-F and *setB*-*mCh*-*pyrG*-R primers) and a 1155 bp 3’UTR (with *setB*-*mCh*-p3 and *setB*-*mCh*-p4 primers) was amplified with *setB*-*mCh*-p7 and *setB*-*mCh*-p8 primers. The fusion PCR product was transformed into the PTS Δku70 (*pyrG*-) strain.

(**B**) The location of SetB in the germinating spore and hyphae shown by DIC imaging (1^st^ column), the location of the nucleus was shown with DAPI staining (2^nd^ column), the location of *SetB-mCherry* (3^rd^ column), and merged photo of nucleus and *SetB-mCherry* (4^th^ column).

**(C)** The subcellular location of SetB under the stress of SDS.


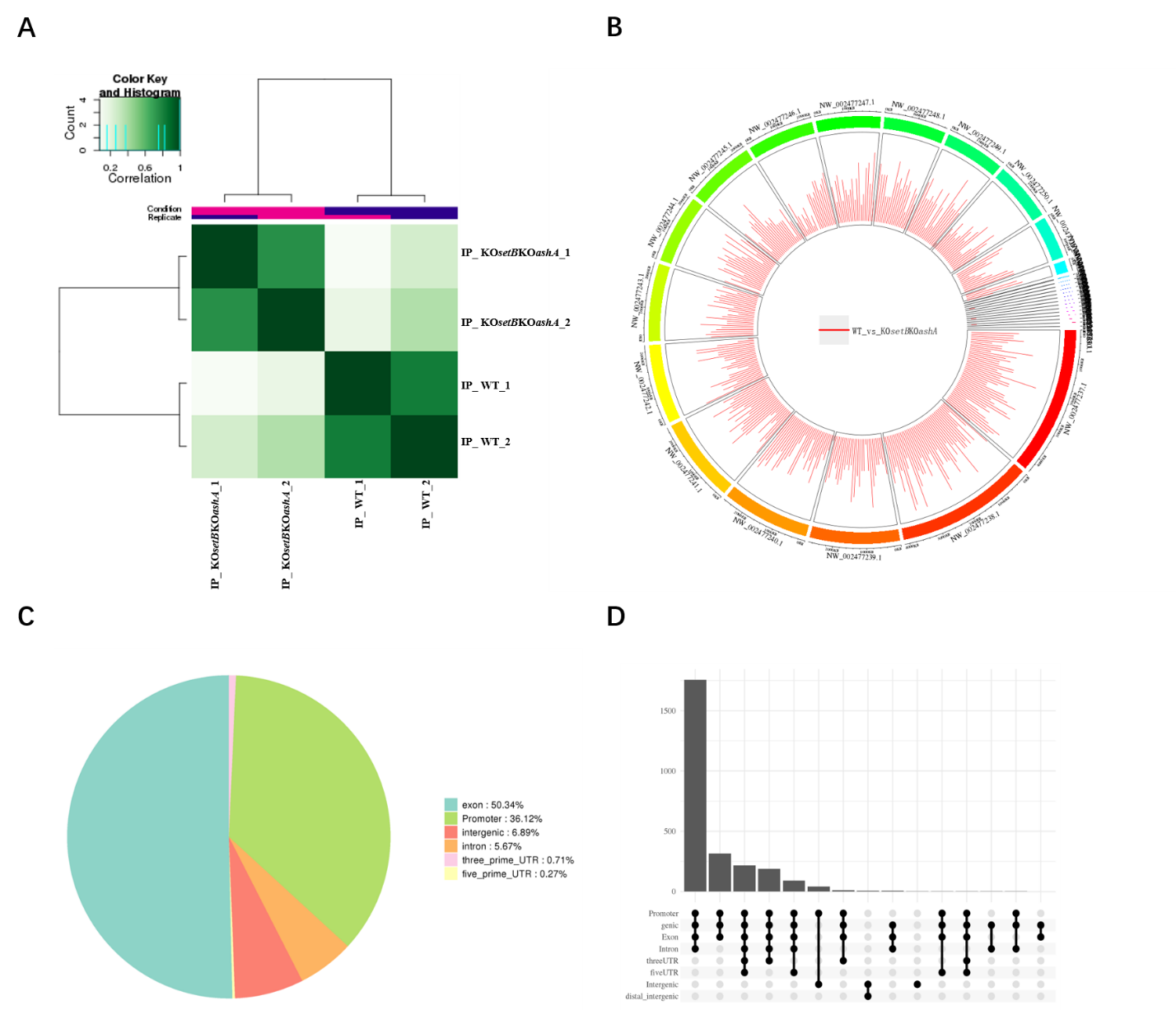


**Figure S7. Trimethylation of H3K36me3 across the whole genome of *A. flavus* was regulated by AshA and SetB.**

**(A**) The heat map of ChIP-seq analysis showing the quality of two repeats of WT samples are correlated, and so do the two repeats of *ΔsetB/ashA* strain samples.

(**B**) The up-peak (WT versus *ΔsetB/ashA* strain) number and their distribution on the whole genome of *A. flavus*.

(**C**) Pie chart showing ratio of the chromatin distribution (including promoter, exon, intron and intergenic region) for the enriched peaks in WT strain compared to *ΔsetB/ashA* strain.

**(D**) Column showing the constitution of up-peaks (WT versus *ΔsetB/ashA* strain).
