# Supplementary material for: Set2 family regulates mycotoxin metabolism and virulence via H3K36 methylation in pathogenic fungus Aspergillus flavus
Source: Virulence. 2022 Aug 9;13(1):1358–78. doi: 10.1080/21505594.2022.2101218 (PMC9364737; doi:10.1080/21505594.2022.2101218)
Supplement: Supplemental Material [file KVIR_A_2101218_SM8872.zip › supplementary/Supplementary Table 1.Primers used in this study.docx]

**Supplementary Table 1. Primers used in this study**

| **Primer name** | **Sequence (5’ → 3’)** | **Fragment amplified** | **Length** |
| --- | --- | --- | --- |
| *ash1*-p1 | CAGTAACGATCCTCGCAATTACTCCATG | 5’-UTR of *ash1* (with *ash1*-p2) | 1.2 kb |
| *ash1*-p2 | TCGTGCGTGTGCTTATTGAGGTCT |  |  |
| *ash1*-p3 | TTTTGGGCTCGTTGGCTACATAGGAATAC | 3’-UTR of *ash1* (with *ash1*-p4) | 1.2 kb |
| *ash1*-p4 | AGCCTCTCCAGGAGGATGAACCTG |  |  |
| *ash1*-p5 | AGACCTCAATAAGCACACGCACGAGCCTCAAACAATGCTCTTCACCC | *A. fumigatus pyrG* (with *ash1*-p6) | 1.9 kb |
| *ash1*-p6 | GTATTCCTATGTAGCCAACGAGCCCAAAAGTCTGAGAGGAGGCACTGATGC |  |  |
| *ash1*-p7 | TCTGGCATCAGCCATCATTAGTTTCTCTCT | nesting primers for fusion PCR of 5’-UTR, 3’-UTR of *ash1* and *A. fumigatus pyrG* | 4.2 kb |
| *ash1*-p8 | TACAGAAGATGCCCAGCCTAGACCCA |  |  |
| *ash1*-p10 | ATGGCCTTTGAGTATTCAGAACAACTCCTC | a fragment from *ash1* | 1 kb |
| *ash1*-p11 | AAGGGAGCGGAGAAAAGATATCGAAGG |  |  |
| *ash1*-p12 | TCCCTTCTGGTCAGCCCAAAC | a fragment from *ash1* | 0.59 kb from G-DNA, 0.48 kb from C-DNA |
| *ash1*-p13 | TAGAGCCCCTGGTAGCATCGA |  |  |
| *pyrG*-R | CAGGAGTTCTCGGGTTGTCG | with *ash1*-p1 to test if *ash1* has been deleted. | 2kb |
| *pyrG*-F | ATCGGCAATACCGTCCAGAAGC | with *ash1*-p4 to test if *ash1* has been deleted | 2,165 kb |
| Com-*ash1*-F | AAAAAAAAAAGCGGCCGCCAGTAACGATCCTCGCAATTACTCCATG | to amplify 5’-UTR - *ash1*- 3’-UTR with Com-*ash1*-R | 5 kb |
| Com-*ash1*-R | AAAAAAAAAAGCGGCCGCAGCCTCTCCAGGAGGATGAACCTG |  |  |
| 5-*pyrG* | GGGTGAAGAGCATTGTTTGAGGCTCGTGCGTGTGCTTATTGAGGTCT | to amplify 5’-UTR with *ash1*-p1 in fusion PCR for Ash1 location detection | 1.21 kb |
| *pyrG*-F1: | GCCTCAAACAATGCTCTTCACCC | to amplify *pyrG* gene with pyrG-R1 in fusion PCR for Ash1 location detection | 1.89 kb |
| *pyrG*-R1 | GTCTGAGAGGAGGCACTGATGC |  |  |
| *gpdA*-F | GCATCAGTGCCTCCTCTCAGACGAGGACTGCAATCGCCATGAGGTTT | to amplify *gpdA* promoter with *gpdA*-R in fusion PCR for Ash1 location detection | 1.51 kb |
| *gpdA*-R | CAAGCTGCGATGAAGTGGGAAAG |  |  |
| *mC*-F | CTTTCCCACTTCATCGCAGCTTGATGGTGAGCAAGGGCGAGG | to amplify *mCherry* with *mC*-R primer in fusion PCR for Ash1 location detection | 0.71 kb |
| *mC*-R | CTTGTACAGCTCGTCCAT |  |  |
| *ash1*-*mC*-F | ATGGACGAGCTGTACAAGATGGCCTTTGAGTATTCA | to amplify front fragment of *ash1* gene with *ash1*-*mC*-R primer in fusion PCR for Ash1 location detection | 1.04 kb |
| *ash1*-*mC*-R | TGACCAGAAGGGAGCGGAGAAA |  |  |
| PTR-F | AAAAAAAAAAGCGGCCGCCCTCTAGAGTCGACCTGCAGGCAT | used in complementation strain preparation |  |
| PTR-R | AAAAAAAAAAGCGGCCGCATCCCCGGGTACCGAGCTCGAATT |  |  |
| qPCR-*ash1*-F | ACACGACGGCAGGTGAAGA | to amplify a fragment from *ash1* in q-PCR analysis | 0.17 kb |
| qPCR-*ash1*-R | TCTCAGATGGACGGCTTAGTT |  |  |
| *tubulin*- F | TTGAGCCCTACAACGCCACT | as internal standard in q-PCR analysis | 0.14 bp |
| *tubulin*- R | TGGTTCAGGTCACCGTAAGAGG |  |  |
| SET-p1F | TCGCTCGGAGACTTCCAGCAACAATG | To amplify the upstream sequence of SET domain in *ash1*^ΔSET^ strain construction | 1300 bp |
| SET-p1R | TCCAATATTGTATTTTCCGCCAGCCTTGG |  |  |
| SET-p2F | CCAAGGCTGGCGGAAAATACAATATTGGA GTAAGGGTACCTTACCGTGTAAATCGGGATTG | To amplify the downstream sequence of SET domain in *ash1*^ΔSET^ strain construction | 810 bp |
| SET-p2R | GGGTGAAGAGCATTGTTTGAGGC GTATTCCTATGTAGCCAACGAGCCCAAAA |  |  |
| *ash1*-p4-F | GCATCAGTGCCTCCTCTCAGACGACAATCCCTGCTAGTTCTTCCCCTCC | To amplify 3’-UTR with *ash1*-p4 in *ash1*^ΔSE^T strain construction | 1100 bp |
| *ash1*-p8-F | CATGGAGTCCTGGAGTGAAGCAACAC | Nesting primer for fusion PCR with p8 in *ash1*^ΔSE^T strain construction | 4760 bp |
| SET-N-R | ACAATGACCTGGGCAGGCTCGAATG | To amplify the upstream sequence of N455A point mutation site with SET-p1F in *ash1*^N455A^ strain construction | 1420 bp |
| SET-N-2F | CATTCGAGCCTGCCCAGGTCATTGT | To amplify the downstream sequence of N455A point mutation site in *ash1*^N455A^ strain construction | 1160 bp |
| SET-N-2R | GGGTGAAGAGCATTGTTTGAGGCGTATTCCTATGTAGCCAACGAGCCCAAAA |  |  |
| SET-N-NF | TCAGACGCTCGTCTCGTTTGAGTCTG | Nesting primer for fusion PCR with p8 in *ash1*^N455A^ strain construction | 5100 bp |
| SET-V-R | GTGTACTCCACAATGTCCTGGTTAGGCTC | To amplify the upstream sequence of V457D point mutation site with SET-p1F in *ash1*^V457D^ strain construction | 1430 bp |
| SET- V -2F | GAGCCTAACCAGGACATTGTGGAGTACAC | To amplify the downstream sequence of V457D point mutation site with SET-N-2R in *ash1*^V457D^ strain construction | 1160 bp |
| *set2*-p1 | CATGTTCGAGGCAACACCCGTTCATG | For 5’-UTR amplification in *Δset2* strain construction | 1.657 kb |
| *set2*-p2 | CGGTTATTGAAGCTAGTAGGCGGATGC |  |  |
| *set2*-p3 | ACGGGAATATCGAGCTACCCCCTTT | For 3’-UTR amplification in *Δset2* strain construction | 1.259 kb |
| *set2*-p4 | GAAACGTCATCTGCTGCAACGACCA |  |  |
| *set2*-p5 | GCATCCGCCTACTAGCTTCAATAACCGGCCTCAAACAATGCTCTTCACCC | For *pyrG* amplification in *Δset2* strain construction | 1.942 kb |
| *set2*-p6 | AAAGGGGGTAGCTCGATATTCCCGTGTCTGAGAGGAGGCACTGATGC |  |  |
| *set2*-p7 | GAGGCCTATCCGGTACATTTGGGCCAC | For fragment fusion in *Δset2* strain construction | 4.261 kb |
| *set2*-p8 | AGTATCGCCGACGTCCTCGGTGTCGAA |  |  |
| *set2*-p9 | ATGTCCCCTCATGACTACGCGGACC | Fragment inside *set2* ORF | 1.726 kb |
| *set2*-p10 | ACACGCTCGTCACCACAGCTCGTCA |  |  |
| P1020 | ATCGGCAATACCGTCCAGAAGC | *pyrG* testing primers, for *Δset2* strain verification |  |
| P801 | CAGGAGTTCTCGGGTTGTCG |  |  |
| Probe-F | AGTCCACGGCTTCCCTAC | Probe in southern-blotting analysis | 0.830 kb |
| Probe-R | GCCTCCATACGCTTCTAAT |  |  |
| Q-*set2*-F | CGACAAAGCCGTCCTAAAGCATCGAG | to amplify a fragment from *set2* in q-PCR analysis | 0.182 kb |
| Q-*set2*-R | CTTCTGCAGTCATGGGAGTGTCTTTCTC |  |  |
| *set2*-C-p3 | GCATCAGTGCCTCCTCTCAGACGTCCGTACTTCTTTAAACATGCTTCGC | 3’-UTR amplification in *Com-Δset2* strain preparation | 1.128 kb |
| *set2*-C-p4 | GAAACGTCATCTGCTGCAACGACCA |  |  |
| *set2*-C-p7 | ACCAGACCAACTAAGCCAGCGCCTCA | For fusion PCR in *Com-Δset2* strain construction | 4.076 kb |
| *set2*-C-p8 | AGTATCGCCGACGTCCTCGGTGTCGAA |  |  |
| *set2*-*mCh*-p1  *set2*-*mCh*-p2 | CCTCAGCCTCCTCCACCTCCAAAAG  CCTCGCCCTTGCTCACCATTACCTTCCCTTCGATCTCAGGTTGAGATGATTT | For 5’-UTR amplification in *set2*-*mCherry* strain construction | 1.061 kb |
| *set2*-*mCh*-F | ATGGTGAGCAAGGGCGAGG | For *mCherry* amplification in *set2*-*mCherry* strain construction | 0.711 kb |
| *set2*-*mCh*-R | CTACTTGTACAGCTCGTCCAT |  |  |
| set2-*mCh*-*pyrG*-F | ATGGACGAGCTGTACAAGTAGGCCTCAAACAATGCTCTTCACCC | For *pyrG* amplification in *set2*-*mCherry* strain construction | 1.89 kb |
| *set2*-*mCh*-*pyrG*-R | GTCTGAGAGGAGGCACTGATGC |  |  |
| *set2*-*mCh*-p3 | GCATCAGTGCCTCCTCTCAGACCTCGGGTCTAGCAGGTTCTAATACTTTGTCC | For 3’-UTR amplification in *set2*-*mCherry* strain construction | 1.155 kb |
| *set2*-*mCh*-p4 | GAAACGTCATCTGCTGCAACGACCA |  |  |
| *set2*-*mCh*-p7 | CAATTCCCGTTAAGAAGGAGGGACAGGAGAAATG | For fusion PCR in *set2*-*mCherry* strain construction | 4.48 kb |
| *set2*-*mCh*-p8 | AGTATCGCCGACGTCCTCGGTGTCGAA |  |  |
| *set2*-pSET1 | CTCACTCTCTTTCCCCCTCATCGCTTC | For 5’UTR amplification in *set2*^ΔSET^ strain construction | 1.013 kb |
| *set2*-pSET1-R | CTGGGCATACTCCTTTCGTTGGAATCTCTG |  |  |
| *set2*-pSET2 | CAGAGATTCCAACGAAAGGAGTATGCCCAG CCGAATGCTACCGCACAAGCTGTCAATC | For 3’UTR amplification in *set2*^ΔSET^ strain construction | 1.364 kb |
| *set2*-pSET2-R | GGGTGAAGAGCATTGTTTGAGGCTCATACCTTCCCTTCGATCTCAGGTTGAGATG |  |  |
| *set2*-pSET4-F | GCATCAGTGCCTCCTCTCAGACCTCGGGTCTAGCAGGTTCTAATACTTTGTCC | 3’UTR amplification in *set2*^ΔSET^ strain construction | 1.155 kb |
| *set2*-p4 | GAAACGTCATCTGCTGCAACGACCA |  |  |
| *set2*-pSET8-F | CCTACTACCACGTCACACAAACACCCTCTGC | Nesting primers in *set2*^ΔSET^ construction | 5.136 kb |
| *set2*-p8 | AGTATCGCCGACGTCCTCGGTGTCGAA |  |  |
| *set2*-PM36-1 | GGAATCACGCGCTAGCAAGAGGATCGACCAATG | For 5’UTR amplification in *set2*^H3K36^ construction | 0.889 kb |
| *set2*-PM36-2 | GTAACGGTGAGGCTTCGCGACACCTCCGGTAG |  |  |
| *set2*-PM36-3 | CTACCGGAGGTGTCGCGAAGCCTCACCGTTAC | Primers for H3K36 amplification | 0.727 kb |
| *set2*-PM36-4 | GGGTGAAGAGCATTGTTTGAGGCCAGCGCGAGATTAAGTCACGACAGTAATGAATTGG |  |  |
| *set2*-PM36-5 | GCATCAGTGCCTCCTCTCAGACAGCGACGCGAACTGTTGAGCGTGTGTA | For 3’UTR amplification in *set2*^H3K36^ construction | 0.885 kb |
| *set2*-PM36-6 | GCAGCAACCCAATCAACAAAACACCAGCCAATC |  |  |
| Δ*set2*Δ*ash1*-p1 | CAGTAACGATCCTCGCAATTACTCCATG | For 5’UTR amplification in *Δset2Δash1* strain construction | 1.212 kb |
| *Δset2*Δ*ash1*-p2 | TCGTGCGTGTGCTTATTGAGGTCT |  |  |
| *Δset2Δash1-*p3 | TTTTGGGCTCGTTGGCTACATAGGAATAC | For 3’UTR amplification in *Δset2Δash1* construction | 1.293 kb |
| *Δset2Δash1-*p4 | AGCCTCTCCAGGAGGATGAACCTG |  |  |
| *Δset2ΔAsh1-PTR*-F | AGACCTCAATAAGCACACGCACGATTAGTGCTTTACGGCACCTCG | For PTR amplification in *Δset2Δash1* construction | 2.926 kb |
| *Δset2ΔAsh1-PTR*-R | GTATTCCTATGTAGCCAACGAGCCCAAAA ACTTTATCCGCCTCCATCCAG |  |  |
| *Δset2Δash1-*p7 | TCTGGCATCAGCCATCATTAGTTTCTCTCT | Nesting primers in *Δset2Δash1* construction | 5.264 kb |
| *Δset2Δash1*-p8 | TACAGAAGATGCCCAGCCTAGACCCA |  |  |
| *Δset2Δash1-*p9 | ATGGCCTTTGAGTATTCAGAACAACTCCTC | The amplification of a fragment inside *ash1* ORF | 1.033 kb |
| *Δset2Δash1-*p10 | AAGGGAGCGGAGAAAAGATATCGAAGG |  |  |
| *wetA-P-1-F* | CCTTACCCATCTTTAGAGCACTT | To amplified the first fragment in the promoter of *wetA* gene in the ChIP-qPCR analysis | 103 bp |
| *wetA-P-1-R* | TGTGTAGGAGTGGTTTGATGGAT |  |  |
| *wetA-P-2-F* | TGTCAATGAATGTGGGCAAG | To amplified the second fragment in the promoter of *wetA* gene in the ChIP-qPCR analysis | 167 bp |
| *wetA-P-2-R* | TGCTTGACCTGAATGCCTG |  |  |
| *wetA-g-F* | ACCCATCATCTCCACCTCCA | To amplified the fragment in the coding sequence of *wetA* gene in the ChIP-qPCR analysis | 142 bp |
| *wetA-g-R* | AACAGCAGAGGGATGAACGG |  |  |
| *aflR-P-1-F* | AAAAGTCGCTGAGAATACGGGT | To amplified the first fragment in the promoter of *aflR* gene in the ChIP-qPCR analysis | 137 bp |
| *aflR-P-1-R* | CGATGCTGACGAAAGATAAAAAG |  |  |
| *aflR-P-2-F* | CGTAAACAAGGAACGCACAG | To amplified the second fragment in the promoter of *aflR* gene in the ChIP-qPCR analysis | 105 bp |
| *aflR-P-2-R* | TTTCGTTGCCTCGCACTTA |  |  |
| *aflR-g-F* | GAATCAACCACCACACGCTC | To amplified the fragment in the coding sequence of *aflR* gene in the ChIP-qPCR analysis | 144 bp |
| *aflR-g-R* | GAAGACAGGGTGCTTTGCTC |  |  |
